# Adaptive MARL-Assisted Hybrid Bat-Artificial Bee Colony Optimization for Energy-Efficient Clustering and Routing in IoT-Enabled Wireless Sensor Networks

**DOI:** 10.3390/s26154996

**Published:** 2026-08-06

**Authors:** H. S. Mohammed, Poria Pirozmand, Sheeraz Memon, Sajad Ghatrehsamani, Sweta Thakur, Rajan Kadel, Bellal Hossain

**Affiliations:** 1 National Academy of Professional Studies (NAPS), Sydney 2010, Australia; bellal.hossain@naps.edu.au; 2 Holmes Institute Sydney, Sydney 2000, Australia; jpirozmand@holmes.edu.au; 3 Melbourne Institute of Technology (MIT), Sydney 3000, Australia; smemon@academic.mit.edu.au; 4 IT Department, King’s Own Institute (KOI), Sydney 2000, Australia; sajad.ghatrehsamani@koi.edu.au (S.G.); sweta.thakur@koi.edu.au (S.T.); 5 National Academy of Professional Studies (NAPS), Melbourne 3000, Australia; rajan.kadel@naps.edu.au

**Keywords:** wireless sensor networks, internet of things, Multi-Agent Reinforcement Learning, Bat Algorithm, Artificial Bee Colony, energy efficiency, cluster-head selection, routing optimization

## Abstract

Energy efficiency remains a major challenge in IoT-enabled wireless sensor networks because sensor nodes operate with limited battery capacity and are often deployed in environments where battery replacement is impractical. Existing clustering and routing protocols frequently optimize cluster-head selection and routing separately, leading to uneven energy consumption, premature node failure, increased routing overhead, and reduced network reliability. This paper proposes an Adaptive Multi-Agent Reinforcement Learning-Assisted Hybrid Bat-Artificial Bee Colony (MARL-BA-ABC) framework for joint cluster-head selection and routing optimization in IoT-enabled wireless sensor networks. The proposed framework combines the Bat Algorithm for local exploitation, the Artificial Bee Colony algorithm for global exploration, and Multi-Agent Reinforcement Learning for adaptive routing. Cluster-head selection and routing are jointly optimized using residual energy, communication distance, traffic load, node density, and link quality. A multi-objective optimization model is formulated to minimize energy consumption, end-to-end delay, routing overhead, and load imbalance while improving packet delivery ratio, residual energy preservation, and network lifetime. Simulation results show that the proposed MARL-BA-ABC framework outperforms Low-Energy Adaptive Clustering Hierarchy (LEACH), Hybrid Energy-Efficient Distributed Clustering (HEED), Particle Swarm Optimization (PSO), Ant Colony Optimization (ACO), Bat Algorithm (BA), and Artificial Bee Colony (ABC), achieving a First Node Death of 2200 rounds, residual energy of 1.32 J, packet delivery ratio of 98.1%, throughput of 410 kbps, and average end-to-end delay of 7.4 ms.

## 1. Introduction

The Internet of Things (IoT) has emerged as one of the most influential technological paradigms of the digital era, enabling seamless interconnection among billions of intelligent devices that cooperate to provide sensing, communication, and autonomous decision-making across diverse application domains [1]. Wireless Sensor Networks (WSNs) constitute a fundamental communication infrastructure within IoT ecosystems by supporting distributed environmental sensing, real-time monitoring, data acquisition, and intelligent information exchange [2]. Consequently, WSNs have become indispensable in numerous IoT applications, including smart cities, intelligent transportation systems, precision agriculture, healthcare monitoring, industrial automation, structural health monitoring, and environmental surveillance, where reliable, scalable, and energy-efficient communication is essential [3]. A typical WSN comprises many spatially distributed sensor nodes equipped with sensing, processing, wireless communication, and limited energy resources that collaboratively collect environmental information and forward the acquired data to a base station through single-hop or multi-hop communication mechanisms [4]. Owing to their constrained battery capacity, limited computational capability, and restricted memory resources, energy consumption remains the primary factor limiting network lifetime and long-term operational sustainability [5]. Since battery replacement or recharging is often impractical in large-scale, hazardous, or inaccessible deployment environments, improving energy efficiency has become one of the most important research challenges in IoT-enabled WSNs [3]. Among the various energy-consuming operations performed by sensor nodes, wireless communication accounts for the largest proportion of total energy expenditure [6]. Consequently, clustering and routing have been extensively investigated as effective strategies for reducing communication overhead, balancing energy consumption among sensor nodes, improving network scalability, and extending network lifetime [7]. In clustering-based architectures, sensor nodes are organized into logical groups, where cluster heads perform local data aggregation before forwarding compressed information to the base station, thereby reducing redundant transmissions and communication costs [8]. Therefore, efficient cluster-head selection and routing-path optimization play a critical role in improving residual energy preservation, packet delivery ratio, communication reliability, end-to-end delay, and overall network lifetime [9]. Traditional clustering protocols, including Low-Energy Adaptive Clustering Hierarchy (LEACH), Hybrid Energy-Efficient Distributed Clustering (HEED), and Power-Efficient Gathering in Sensor Information Systems (PEGASIS), introduced energy-aware communication mechanisms that significantly improved network efficiency compared with direct transmission approaches [10]. Nevertheless, these protocols generally rely on predefined, probabilistic, or static decision-making mechanisms, which restrict their ability to adapt to dynamic network conditions, including variations in residual energy, traffic load, node density, and communication quality [10]. To overcome these limitations, numerous metaheuristic optimization algorithms, including Particle Swarm Optimization (PSO), Ant Colony Optimization (ACO), Artificial Bee Colony (ABC), Grey Wolf Optimizer (GWO), and the Bat Algorithm (BA), have been employed to optimize cluster-head selection and routing decisions [7,8,11,12]. Although these approaches demonstrate strong global optimization capability and improved energy balancing, they may still suffer from premature convergence, sensitivity to parameter settings, local-optimum stagnation, and reduced adaptability under highly dynamic network environments [11]. More recently, reinforcement learning (RL) has emerged as a promising paradigm for adaptive routing and intelligent network optimization by enabling sensor nodes to continuously improve communication decisions through interaction with the network environment [11,12]. Unlike conventional optimization approaches that repeatedly solve a static optimization problem, reinforcement learning enables routing policies to evolve dynamically according to observed network states, thereby improving adaptability under changing traffic conditions, residual energy distribution, and link quality. Although the previously proposed BA-ABC framework [12] demonstrated that integrating the Bat Algorithm and Artificial Bee Colony algorithm could improve energy-efficient clustering and routing performance in WSNs, its optimization strategy remained entirely metaheuristic and relied on fixed interactions between the two optimization components. Consequently, routing decisions were generated through repeated optimization without incorporating any adaptive learning mechanism capable of responding continuously to dynamic changes in network conditions. Variations in residual energy, traffic load, congestion, wireless link quality, or network topology therefore required repeated optimization cycles, which may reduce adaptability and increase computational overhead in dynamic IoT-enabled WSN environments. To overcome these limitations, this study proposes a Multi-Agent Reinforcement Learning-assisted Bat Algorithm–Artificial Bee Colony (MARL-BA-ABC) framework that integrates distributed reinforcement learning with hybrid metaheuristic optimization within a unified optimization architecture. In the proposed framework, each cluster head operates as an autonomous learning agent that continuously adapts next-hop routing decisions according to real-time observations of residual energy, communication distance, traffic load, neighbourhood density, congestion level, and wireless link quality. Unlike the previous BA-ABC framework, where the contributions of BA and ABC remain fixed throughout the optimization process, the proposed framework introduces an adaptive reward-driven fusion mechanism that dynamically adjusts the relative influence of BA, ABC, and MARL according to the evolving network state. Furthermore, cluster-head selection and multi-hop routing are optimized jointly through a unified multi-objective optimization model, enabling routing feedback to influence clustering decisions while clustering outcomes simultaneously constrain feasible routing actions. This bidirectional interaction improves energy balancing, routing stability, packet delivery performance, throughput, communication latency, and overall network lifetime. Consequently, the proposed MARL-BA-ABC framework represents a significant methodological advancement over the earlier BA-ABC approach by transforming a static hybrid metaheuristic optimizer into an adaptive, state-aware, and learning-assisted clustering–routing framework suitable for highly dynamic IoT-enabled WSN environments. Despite considerable advances in clustering protocols, metaheuristic optimization, and reinforcement-learning-based routing, existing studies generally address these components independently. Conventional clustering protocols typically lack adaptive learning capability, metaheuristic optimization methods provide limited responsiveness to dynamic network conditions, and reinforcement-learning-based approaches often focus primarily on routing while overlooking energy-aware cluster-head optimization [7]. Consequently, there remains a need for an integrated framework capable of jointly combining adaptive learning, global exploration, local exploitation, energy-aware clustering, and intelligent routing within a unified optimization architecture. To address this research gap, this study makes five principal contributions. First, it proposes a novel MARL-BA-ABC framework that jointly optimizes cluster-head selection and multi-hop routing for IoT-enabled wireless sensor networks. Second, it introduces an adaptive reward-driven fusion mechanism that dynamically balances the contributions of BA, ABC, and MARL according to network-state feedback and optimization performance. Third, it formulates a unified multi-objective optimization model that simultaneously maximizes network lifetime, residual energy, and packet delivery ratio while minimizing energy consumption, communication overhead, load imbalance, and end-to-end delay. Fourth, extensive simulations are conducted using Python and NS-3 under representative smart-city and healthcare IoT scenarios to evaluate network lifetime, routing reliability, scalability, convergence behaviour, and overall communication performance. Finally, a statistical validation framework based on confidence intervals, one-way analysis of variance (ANOVA), Wilcoxon rank-sum testing, and effect-size analysis is incorporated to facilitate rigorous performance assessment when complete run-level statistical outputs are available. The remainder of this paper is organized as follows. Section 2 reviews existing clustering protocols, metaheuristic optimization methods, and reinforcement-learning-based routing approaches for IoT-enabled WSNs. Section 3 presents the network model, energy-consumption model, clustering formulation, routing model, and multi-objective optimization framework. Section 4 describes the proposed MARL-BA-ABC algorithm, including BA-based local exploitation, ABC-based global exploration, MARL-assisted adaptive routing, and the adaptive reward-driven fusion mechanism. Section 5 presents the simulation environment, benchmark algorithms, evaluation metrics, parameter settings, and statistical validation procedure. Section 6 discusses the experimental results, convergence analysis, ablation study, deployment considerations, practical implications, limitations, and threats to validity. Finally, Section 7 concludes the paper and outlines future research directions.

## 2. Related Work

This section reviews the main research streams related to the proposed framework. First, traditional clustering protocols are discussed to identify the limitations of static and probabilistic cluster-head selection. Second, metaheuristic optimization approaches are examined to highlight their strengths in search-space exploration and exploitation, as well as their limitations in dynamic WSN environments. Third, reinforcement-learning-based routing approaches are reviewed to clarify how adaptive decision-making can improve routing performance. The section concludes by identifying the research gap that motivates the proposed MARL-BA-ABC framework.

### 2.1. Traditional Clustering Protocols

Energy-efficient clustering and routing have long been recognized as fundamental challenges in WSN and IoT-enabled sensing environments. Among the earliest and most influential clustering protocols, LEACH introduced a distributed cluster-head rotation mechanism to balance energy consumption among sensor nodes and reduce communication overhead [8]. By randomly rotating cluster-head responsibilities, LEACH significantly extended network lifetime compared with direct communication approaches. However, the probabilistic cluster-head selection strategy often results in uneven cluster distribution and suboptimal energy utilization under dynamic network conditions [8]. To address some of the limitations of LEACH, HEED protocol incorporated residual energy and communication cost metrics into the cluster-head selection process. HEED achieves improved cluster stability and load balancing while reducing the likelihood of selecting low-energy nodes as cluster heads. Nevertheless, the protocol may still converge to locally optimal solutions and exhibits limited adaptability to large-scale heterogeneous IoT environments [8,9]. Another widely adopted hierarchical routing protocol is PEGASIS, which organizes sensor nodes into communication chains to minimize long-distance transmissions. Although PEGASIS reduces overall energy consumption and improves network lifetime, the chain-based communication structure introduces additional transmission delays and scalability challenges, particularly in dense IoT deployments characterized by dynamic traffic patterns and heterogeneous devices [8,12].

### 2.2. Metaheuristic Optimization Approaches

To overcome the limitations of traditional clustering protocols, numerous studies have employed metaheuristic optimization techniques for cluster-head selection and routing optimization. Particle Swarm Optimization (PSO) has been extensively utilized due to its simplicity and fast convergence characteristics. PSO-based clustering methods can effectively optimize energy consumption and communication distance; however, they often suffer from premature convergence and reduced exploration capabilities in complex search spaces [13]. ACO has also attracted considerable attention for routing optimization because of its distributed search mechanism and ability to discover efficient communication paths. Despite its effectiveness, ACO frequently experiences slow convergence and high computational overhead in large-scale networks [7]. More recently, swarm-intelligence algorithms such as ABC, BA, GWO, and Harris Hawks Optimization (HHO) have demonstrated promising results for multi-objective WSN optimization. ABC provides strong global exploration capabilities but may exhibit slow convergence during later optimization stages. BA offers efficient local exploitation and rapid convergence but remains vulnerable to local optima. Similarly, GWO and HHO achieve effective exploration–exploitation balance; however, their performance is highly dependent on parameter tuning and problem-specific configurations. Consequently, hybrid metaheuristic frameworks have been proposed to exploit the complementary strengths of multiple optimization algorithms while mitigating individual limitations [7].

### 2.3. Reinforcement Learning-Based Approaches

Recent studies have increasingly explored reinforcement learning (RL) and MARL frameworks for intelligent routing and resource allocation in IoT-enabled wireless sensor networks because they enable adaptive decision-making under dynamic network conditions and uncertain environments [13]. Q-learning-based routing protocols enable sensor nodes to learn optimal forwarding policies directly through interaction with the network environment without requiring prior knowledge of network dynamics. Such approaches have demonstrated improved energy efficiency, routing adaptability, and packet delivery performance under changing traffic and topology conditions [14]. Similarly, Deep reinforcement learning has further improved routing performance by enabling adaptive policy learning in high-dimensional state spaces and has recently been applied to IoT and WSN routing to reduce delay, improve packet delivery ratio, and extend network lifetime [15]. However, most existing reinforcement-learning-based approaches focus primarily on routing optimization while overlooking cluster-head selection. More recently, MARL has attracted considerable attention because multiple agents can cooperatively optimize routing decisions using local observations while improving scalability, load balancing, and robustness in distributed wireless sensor networks. Nevertheless, relatively few studies integrate MARL with hybrid metaheuristic optimization for simultaneous cluster-head selection and routing optimization [16]. Consequently, the development of unified frameworks that jointly optimize clustering and routing while maintaining energy efficiency and scalability remains an important research challenge.

Table 1 provides a comparative analysis of representative traditional, metaheuristic, and reinforcement-learning-based approaches for WSN clustering and routing optimization. As shown in Table 1, existing methods exhibit important limitations with respect to adaptive learning, joint clustering–routing optimization, global exploration, and local exploitation. Traditional protocols such as LEACH, HEED, and PEGASIS lack learning capability, while metaheuristic approaches including PSO, ACO, BA, ABC, GWO, and HHO often suffer from convergence instability, parameter sensitivity, or limited adaptability to dynamic network conditions [8,11,12]. Although reinforcement learning techniques such as Q-learning and MARL improve adaptive decision-making, they primarily focus on routing optimization and generally neglect cluster-head selection [13,16]. Despite substantial advances in reinforcement learning and methodology, existing approaches generally optimize either routing or cluster-head selection independently, rely on static optimization strategies, or lack adaptive cooperation between learning and evolutionary search mechanisms. Consequently, the integration of MARL with complementary metaheuristic algorithms for joint clustering and routing remains insufficiently explored [17]. This research gap motivates the development of the proposed MARL-BA-ABC framework, which simultaneously combines adaptive learning, energy-aware clustering, intelligent routing, and hybrid optimization to improve network performance and lifetime.

## 3. System Model and Mathematical Formulation

This section presents the mathematical foundation of the proposed framework. It first defines the IoT-enabled WSN topology and communication model, followed by the radio-energy model used to estimate transmission, reception, and aggregation costs. The section then formulates residual-energy dynamics, cluster-head selection, routing optimization, and global multi-objective optimization. Finally, the reinforcement-learning formulation, Bellman optimality equation, hybrid BA-ABC-MARL integration mechanism, and convergence discussion are presented to support the proposed adaptive optimization strategy.

### 3.1. Network Model

Consider an IoT-enabled wireless sensor network (WSN) comprising N stationary sensor nodes randomly deployed over a two-dimensional sensing area. The network topology is modelled as an undirected graph:

(1) G=(V,E)

where $V=\{v_{1},v_{2},\ldots ,v_{N}\}$ denotes the set of sensor nodes and E⊆V×V represents the set of feasible wireless communication links established between neighbouring nodes within their communication range. Each sensor node $v_{i}\in V$ is characterized by its spatial position and residual energy and is represented as

(2) $$v_{i}=(x_{i},y_{i},E_{i}^{res})$$

where $x_{i}$ and $y_{i}$ denote the Cartesian coordinates of node $v_{i}$, and $E_{i}^{res}$ represents its current residual energy. The residual energy is continuously updated during network operation and serves as one of the principal optimization criteria for cluster-head selection and routing decisions throughout the proposed framework. The Euclidean distance between two neighbouring sensor nodes $v_{i}$ and $v_{j}$ is then computed as

(3) $$d_{ij}=\sqrt{{\left(x_{i}-x_{j}{)}^{2}+(y_{i}-y_{j}\right)}^{2}}$$

while the distance between node $v_{i}$ and the base station is calculated as

(4) $$d_{i,BS}=\sqrt{{\left(x_{i}-x_{BS}{)}^{2}+(y_{i}-y_{BS}\right)}^{2}}$$

This ordering first defines the overall network topology and then introduces the individual node representation, providing a clearer and more logical mathematical formulation [17].

### 3.2. First-Order Radio Energy Model

The proposed framework adopts the first-order radio energy dissipation model, which remains widely used in contemporary WSN energy-optimization studies [3]. The transmission energy required for sending an l-bit packet over distance d is given by Equations (5) and (6) [7]:

(5) $$E_{TX}(l,d)\;=\;lE_{elec}\;+\;l\varepsilon_{fs}\;d^{2},\;\;d\;<\;d_{0}$$

(6) $$E_{TX}(l,d)=lE_{elec}+l\varepsilon_{mp}\;d^{4},\;\;d\;\geq \;d_{0}$$

The threshold distance is:

(7) $$d_{0}\;=\;\surd (\varepsilon_{fs}\;/{\;\varepsilon }_{mp})$$

The receiving energy is:

(8) $$E_{RX}(l)\;=\;lE_{elec}$$

The data aggregation energy is:

(9) $$E_{DA}(l)\;=\;lE_{DA}$$

Accordingly, the total cluster-head communication energy becomes:

(10) $$E_{CH}\;=\;E_{RX}\;+\;E_{DA}\;+\;E_{TX}$$

### 3.3. Residual Energy Dynamics

The residual energy of node v_i_ at communication round r is:

(11) $$E_{i}(r+1)\;=\;E_{i}(r)\;-\;E_{i},consumed(r)$$

After R rounds,

(12) $$E_{i}(R)=E_{i}(0)-{\Sigma_{r=1}}^{R}\;E_{i},consumed(r)$$

Assuming continuous energy depletion,

(13a) $$dE_{i}/dr=-E_{i},consumed(r)$$

Integrating both sides yields:

(13b) $$E_{i}(R)\;=\;E_{i}(0)\;-\;{\int_{0}}^{R}\;E_{i},consumed(r)dr$$

Equation (13) represents cumulative energy depletion over time and is based on standard energy conservation principles [18].

### 3.4. Cluster-Head Selection Optimization (Proposed)

To jointly consider energy balance and communication efficiency, the cluster-head fitness function is formulated as:

(14) $$F_{CH\;}=\;w_{1}(1\;-\;E_{res}/E_{max})\;+\;w_{2}(d_{avg}/d_{max})\;+\;w_{3}(d_{CH},BS/d_{max})\;+\;w_{4}(L_{CH}/L_{max})$$

The term $d_{CH},BS$ denotes the distance between the candidate cluster head and the base station. The normalization factor $d_{max}$ denotes the maximum possible communication distance in the deployment area. The proposed cluster-head fitness function jointly evaluates four normalized factors: residual-energy deficit, average intra-cluster communication distance, distance from the candidate cluster head to the base station, and normalized cluster traffic load. The first term discourages the selection of low-energy nodes as cluster heads. The second term favours candidate cluster heads that are centrally located with respect to their assigned member nodes, thereby reducing intra-cluster communication costs. The third term accounts for the forwarding distance from the candidate cluster head to the base station, which directly affects inter-cluster transmission energy. The fourth term penalizes overloaded cluster heads and supports balanced traffic distribution. The weighting coefficients are normalized to prevent any single objective from dominating the decision-making process. Consequently, the resulting cluster-head selection strategy improves energy balance, communication efficiency, and network lifetime and is subject to:

(15) $$w_{1}\;+\;w_{2}\;+\;w_{3}\;+\;w_{4}\;=\;1,\;\;\;\;\;w_{k}\;\geq \;0$$

where:

(16) $$d_{avg}\;=\;(1/N_{CH})\;\Sigma_{j}\in CH\;d(CH,j)$$

(17) $$L_{CH}=\Sigma_{j}\in CH\;traffic(j)$$

where $N_{CH}$ is the number of member nodes assigned to the candidate cluster head, and d(CH,j) is the Euclidean distance between the candidate cluster head and member node j.

### 3.5. Routing Optimization Model (Proposed)

The routing energy cost is defined as the total communication energy consumed along the selected routing path, rather than the sum of residual node energies. Therefore, the energy cost of a candidate path is reformulated as:

(18) $$\mathrm{F\_Route}\;=\;\beta_{1}E_{path}\;+\;\beta_{2}D_{path}\;+\;\beta_{3}H_{path}\;-\;\beta_{4}LQ_{path}$$

where $E_{path}$ denotes the total communication energy consumed along the selected routing path, $D_{path}$ denotes the total routing delay, $H_{path}$ denotes the hop count, and $LQ_{path}$ denotes the average link quality of the selected path. The weighting coefficients $\beta_{1},\;\beta_{2},\;\beta_{3},\;\mathrm{and}\;\beta_{4}$ control the relative importance of energy consumption, delay, hop count, and link reliability, respectively. The energy-cost component is computed as follows:

(19) $$E_{path}\;=\;\Sigma_{i}\in path\;[E_{TX}(i)\;+\;E_{RX}(i)\;+\;E_{DA}(i)]$$

where $E_{TX}(i)$ denotes the energy consumed by sensor node i during packet transmission, $E_{RX}(i)$ denotes the energy consumed during packet reception, and $E_{DA}(i)$ represents the energy required for data aggregation whenever aggregation is performed at intermediate nodes or cluster heads. Accordingly, the path-energy term quantifies the total communication and processing energy expended during packet forwarding rather than the residual energy remaining in the participating nodes. This formulation enables the routing optimization process to accurately evaluate the energy cost associated with end-to-end packet transmission while accounting for both communication and processing overhead:

(20) $$Minimize\;F_{Route}$$

### 3.6. Global Multi-Objective Optimization

The clustering and routing objectives are integrated into a unified optimization framework:

(21) $$F_{Total\;}=\;\lambda_{1}F_{CH}\;+\;\lambda_{2}F_{Route}$$

subject to

(22) $$E_{i}>E_{min}$$

(23) $$PDR\;\geq \;{PDR}_{min}$$

(24) $$Delay\;\leq \;{Delay}_{max}$$

The overall optimization problem is:

(25) $$Minimize\;F_{Total}$$

### 3.7. Lagrangian Optimization Framework

The constrained multi-objective optimization problem is transformed into an unconstrained Lagrangian form by introducing non-negative multipliers for energy, connectivity, load-balancing, and routing constraints:

(26) $$L(x,\lambda )\;=\;F_{Total}(x)\;+\;\Sigma_{i}\;\lambda_{i}g_{i}(x)$$

The corresponding Karush–Kuhn–Tucker conditions comprise primal feasibility, dual feasibility, complementary slackness, and stationarity. Based on nonlinear optimization theory [19], these conditions provide necessary optimality requirements under differentiability and appropriate constraint-qualification assumptions. The KKT conditions provide necessary optimality conditions for the constrained multi-objective formulation under differentiability and constraint-qualification assumptions; however, because the proposed hybrid optimization process is metaheuristic and learning-assisted, practical optimality is assessed through simulation-based convergence behaviour and comparative performance evaluation.

### 3.8. Multi-Agent Reinforcement Learning Formulation

The routing environment is modelled as a Markov Decision Process (MDP) [20]:

(27) $$MDP\;=\;(S_{t},\;A_{t},\;P,\;R_{t},\;\gamma )$$

The reward function is defined as:

(28) $$R\;=\;\alpha_{1}E_{saving}\;+\;\alpha_{2}PDR\;+\;\alpha_{3}LQ\;-\;\alpha_{4}Delay\;-\;\alpha_{5}EnergyCost$$

The objective is:

(29) $$\pi \;*=\;{argmax}_{\pi }\;E[\Sigma_{t}\;\gamma^{t}R_{t}]$$

### 3.9. Bellman Optimality Equation

The optimal action-value function is:

(30) $$Q*(s,a)\;=\;E[R_{t}\;+\;\gamma \;{max}_{a}\prime \;Q*(s\prime ,a\prime )]$$

The Q-learning update rule is:

(31) $$Q(s,a)\;\leftarrow \;Q(s,a)\;+\;\eta [R\;+\;\gamma \;{max}_{a}\prime \;Q(s\prime ,a\prime )\;-\;Q(s,a)]$$

Equations (27)–(31) are based on the classical Markov decision process and Q-learning framework introduced by Watkins and Dayan [21,22].

### 3.10. Hybrid BA-ABC-MARL Optimization Mechanism

The integrated solution is generated as:

(32) $$X_{t+1}\;=\;\rho_{1}X_{BA}\;+\;\rho_{2}X_{ABC}\;+\;\rho_{3}X_{MARL}$$

subject to:

(33) $$\rho_{1}\;+\;\rho_{2}\;+\;\rho_{3}\;=\;1,\;\;\;\;\;\rho_{k}\;\geq \;0$$

Adaptive weights are updated according to:

(34) $$\rho_{k}(t+1)\;=\;\rho_{k}(t)\;+\;\eta (R_{k}\;-\;R_{avg})$$

Equations (32)–(34) constitute the proposed adaptive BA-ABC-MARL fusion mechanism. Unlike conventional hybrid optimization methods that employ fixed contribution weights, the proposed framework introduces an adaptive reward-driven weighting mechanism that dynamically adjusts the influence of the BA, ABC, and MARL components according to real-time optimization performance.

### 3.11. Convergence Analysis

The Bellman operator is defined as

(35) $$TQ(s,a)=R(s,a)+\gamma \;{{max}_{a}}^{\prime }\;Q(s^{\prime },a^{\prime })$$

For any two value functions $Q_{1}\;and\;Q_{2},$

(36) $$||TQ_{1}-TQ_{2}||\infty \;\leq \;\gamma ||Q_{1}-Q_{2}||\infty$$

Since 0 ≤ γ < 1, T is a contraction mapping. By the Banach Fixed Point Theorem [20]:

(37) $${lim}_{t}\to \infty \;Q_{t}\;=\;Q*$$

It should be noted that the convergence discussion applies specifically to the tabular Q-learning component under finite state-action spaces, bounded rewards, and sufficient exploration. Therefore, the Bellman contraction argument does not automatically guarantee global convergence of the complete hybrid MARL-BA-ABC framework. The hybrid metaheuristic layer, which integrates BA exploitation and ABC exploration with reinforcement-learning feedback, is empirically validated through convergence curves and performance stability analysis across repeated simulation runs.

## 4. Proposed MARL-BA-ABC Algorithm Design

This section explains the design and operational workflow of the proposed MARL-BA-ABC framework. The algorithm integrates three complementary components: the Bat Algorithm for local exploitation, the ABC algorithm for global exploration, and Multi-Agent Reinforcement Learning for adaptive routing decisions [21]. The section describes the framework overview, candidate cluster-head generation, global optimization, learning-based routing module, adaptive reward-driven fusion mechanism, joint clustering–routing optimization, algorithmic steps, and computational complexity.

### 4.1. Framework Overview

**Figure 1:**
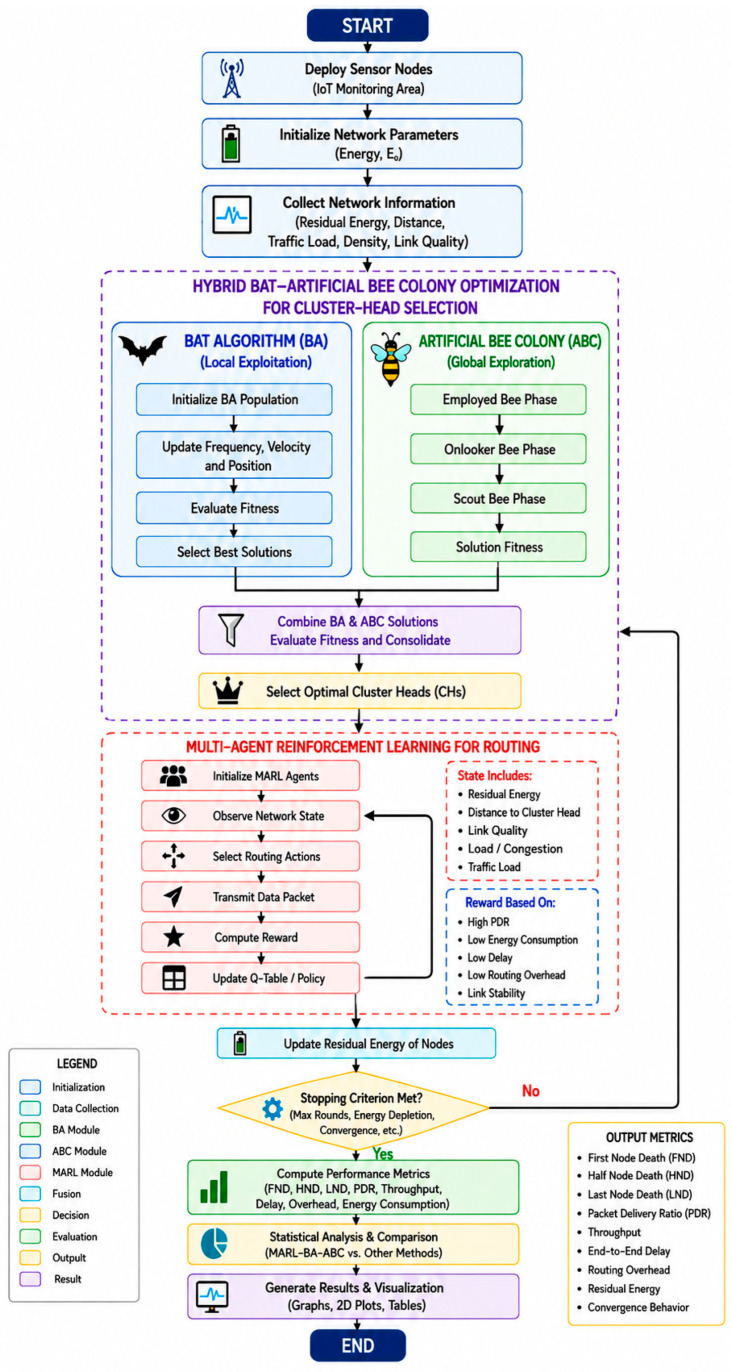
Workflow of the proposed MARL-BA-ABC framework.

Figure 1 illustrates the overall workflow of the proposed MARL-BA-ABC framework. The diagram summarizes the interaction between the Bat Algorithm (BA), Artificial Bee Colony (ABC), and Multi-Agent Reinforcement Learning (MARL) modules for joint cluster-head selection and routing optimization. Detailed operational steps are presented in Algorithm 1 and the corresponding subsections to avoid redundant explanation.

### 4.2. Bat Algorithm-Based Cluster-Head Candidate Generation

The BA Algorithm is employed to generate candidate cluster-head configurations and perform local exploitation within the search space. Let the population of bats be represented by:

(38) $$X_{BA}=\;\{x_{1},\;x_{2},\;\ldots ,\;x_{NB}\}$$

where NB denotes the total number of bats and each solution corresponds to a candidate cluster-head configuration. The frequency update mechanism is defined as:

(39) $$f_{i}\;=\;f_{min}\;+\;(f_{max}\;-\;f_{min})\beta ,\;\;\;\;\;\beta \;\in \;[0,1]$$

where β is a uniformly distributed random number in the interval [0, 1]. The velocity update rule is given by:

(40) $${v_{i}}^{t}\;=\;{v_{i}}^{t-1}\;+\;({x_{i}}^{t-1}\;-\;x*)f_{i}$$

where x* represents the best solution obtained so far. The position update equation is:

(41) $${x_{i}}^{t}\;=\;{x_{i}}^{t-1}\;+\;{v_{i}}^{t}$$

The loudness update mechanism is expressed as

(42) $${A_{i}}^{t+1}=\alpha {A_{i}}^{t}$$

The pulse-emission rate update is given by

(43) $${r_{i}}^{t+1}={r_{i}}^{0}[1-exp(-\gamma t)]$$

where α and γ are control parameters that regulate the exploitation behaviour of the Bat Algorithm. Equations (38)–(43) are adopted from the standard BA [23].

### 4.3. Artificial Bee Colony-Based Global Optimization

To improve exploration capability and avoid local-optimum stagnation, the Artificial Bee Colony algorithm is integrated into the optimization framework. A neighbouring candidate solution is generated as:

(44) $$v_{\mathrm{ij}}\;=\;x_{\mathrm{ij}}\;+\;\phi_{\mathrm{ij}}(x_{\mathrm{ij}}\;-\;x_{\mathrm{kj}}),\;\;\;\;\;k\;\neq \;i$$

where $\phi_{\mathrm{ij}}$ is a random perturbation coefficient. The fitness value of each solution is computed using:

(45) $$f{it}_{i}\;=\;1/(1\;+\;F_{i})$$

where $F_{i}$ denotes the objective function value. The probability of selecting solution i is determined as:

(46) $$p_{i}\;=\;f{it}_{i}\;/\;\Sigma_{j}\;f{it}_{j}$$

When a solution becomes stagnant, the scout-bee phase generates a new candidate solution according to:

(47) $$x_{\mathrm{ij}}\;=\;x_{min},j\;+\;r\;and\;(0,1)(x_{max},j\;-\;x_{min},j)$$

Equations (44)–(47) are based on the classical Artificial Bee Colony optimization framework [11].

### 4.4. Multi-Agent Reinforcement Learning Module

Each cluster head is modelled as an autonomous learning agent operating within a Markov Decision Process. The MARL routing module uses the reward function defined in Equation (28) and updates the action-value function using the Q-learning rule presented in Equation (31). Each agent observes residual energy, base-station distance, traffic load, neighbouring-node density, link quality, and congestion status before selecting a routing action. These state variables allow the agent to evaluate both energy availability and communication reliability before forwarding packets. The action space consists of the feasible routing decisions available to each cluster-head agent. At each communication round, an agent may select a neighbouring cluster head as the next-hop relay, transmit data directly to the base station when the transmission distance is suitable, or avoid a congested or unreliable path. This action design enables the routing process to adapt dynamically to changes in residual energy, link condition, and network congestion. The action-selection process follows an exploration–exploitation strategy. During learning, each agent occasionally explores alternative routing paths to discover better forwarding options while exploiting the best-known routing decision based on previously learned rewards. This policy prevents the framework from relying on a fixed route and helps the network adapt to changing topology, traffic load, and energy conditions. Agent interaction occurs through local network feedback and shared routing conditions. Each cluster-head agent updates its routing decision according to information received from neighbouring nodes, including residual energy, link quality, congestion level, and reward feedback. This cooperative interaction helps the network avoid overloaded routes, balance energy consumption, and maintain reliable packet forwarding. The learning process continues until one of the stopping conditions is satisfied, including reaching the maximum number of communication rounds, the residual network energy falling below a predefined threshold, the absence of a feasible routing path, or negligible improvement in learned routing values across consecutive iterations. The action space consists of all feasible neighbouring cluster-head forwarding options together with the direct transmission action to the base station, yielding $|A_{i}|$ available actions for each learning agent.

### 4.5. Adaptive Reward-Driven Hybrid BA-ABC-MARL Integration Mechanism

The adaptive BA-ABC-MARL integration mechanism operationalizes the mathematical formulation presented in Section 3.8, Section 3.9 and Section 3.10. Specifically, the MARL routing component follows the Markov Decision Process (MDP) and Q-learning formulation defined by Equations (27)–(31), whereas the adaptive fusion mechanism is governed by Equations (32)–(34), which dynamically adjust the relative contributions of the BA, ABC, and MARL components according to reward feedback and current network conditions. During each communication round, BA performs local exploitation around promising cluster-head candidates, ABC enhances global exploration to avoid premature convergence, and MARL adaptively updates routing decisions based on the observed network state. This coordinated interaction enables the proposed framework to balance exploration, exploitation, and adaptive learning while improving convergence stability, energy efficiency, and routing reliability.

### 4.6. Joint Clustering–Routing Optimization

Unlike conventional approaches that optimize clustering and routing independently, the proposed framework performs simultaneous optimization of both processes. The joint clustering–routing objective follows the mathematical formulation introduced in Section 3, Equations (21)–(25). During implementation, cluster-head selection and routing decisions are optimized cooperatively using the adaptive MARL-BA-ABC framework. The cluster-head selection process identifies energy-efficient and well-positioned nodes for data aggregation, while the routing module determines reliable and low-cost forwarding paths based on residual energy, link quality, traffic load, and congestion status. By coupling these two decisions, the proposed framework reduces unnecessary transmissions, balances network load, minimizes routing overhead, and delays node energy depletion. By coupling these two decisions, the proposed framework reduces unnecessary transmissions, balances network load, minimizes routing overhead, and delays node energy depletion. The overall implementation procedure of the proposed MARL-BA-ABC framework is presented in Algorithm 1.
**Algorithm 1:** Proposed MARL-BA-ABC Framework**Input:** Network topology G=(V,E); initial node energy $E_{0}$; BA population size $N_{B}$; ABC population size $N_{A}$; MARL learning parameters ($\alpha ,\;\gamma ,\;\varepsilon_{0}$); maximum communication rounds T.
**Output:** Optimal cluster-head set CH*; optimal routing paths Π*; network lifetime metrics (FND, HND, LND); residual energy; packet delivery ratio; throughput; delay; routing overhead; convergence performance. 1. Initialize the IoT-WSN topology and randomly deploy sensor nodes within the monitoring area. 2. Assign the initial node energy $E_{0}$ to every sensor nodes. 3. Initialize the BA population, ABC food sources, MARL agents, and Q-table. 4. For each communication round r = 1, 2, …, T do 5. Collect network-state information, including residual energy, node distance, node density, traffic load, and link quality. 6. Generate candidate cluster-head solutions using the Bat Algorithm. 7. Update BA frequency, velocity, position, loudness, and pulse-emission rate. 8. Evaluate BA candidate solutions using the cluster-head fitness function. 9. Apply the Artificial Bee Colony algorithm to improve global exploration. 10. Execute the employed-bee, onlooker-bee, and scout-bee phases. 11. Evaluate all ABC candidate solutions. 12. Combine BA and ABC candidate solutions using the hybrid optimization mechanism. 13. Select the optimal cluster-head set CH*. 14. Assign each ordinary sensor node to the nearest optimal cluster head. 15. For each MARL agent associated with a cluster head, do: 16. Observe the current network state $S_{t}$. 17. Select the routing action $A_{t}$ using the exploration-exploitation policy. 18. Forward data packets through the selected next-hop node. 19. Compute the reward R_t based on energy saving, PDR, link quality, delay, and energy cost. 20. Update the Q-value using:        $Q(S_{t},A_{t})\;\leftarrow \;Q(S_{t},A_{t})\;+\;\alpha [R_{t}\;+\;\gamma \;{max}_{a}\;Q(S_{\left\{t+1\right\}},a)\;-\;Q(S_{t},A_{t})]$ 21. End for. 22. Update the residual energy of all transmitting, receiving, and aggregating nodes. 23. Adapt the BA-ABC-MARL contribution weights according to reward feedback. 24. Check node status and update alive/dead node records. 25. If the stopping criterion is satisfied, terminate the optimization process. 26. End for. 27. Compute FND, HND, LND, total residual energy, PDR, throughput, delay, routing overhead, and convergence speed. 28. Return CH*, the optimal routing path, and all performance metrics.

Let $N_{B}$ denote the BA population size, $N_{A}$ the number of ABC food sources, $N_{M}$ the number of MARL agent-state updates performed per communication round, N the number of sensor nodes, and T the number of optimization iterations. If the cost of evaluating each candidate solution is treated as constant, the algorithm-level computational complexity is:

(48) $$O\left[T(N_{B}+N_{A}+N_{M})\right]$$

If each BA and ABC candidate-fitness evaluation examines all N sensor nodes, the implementation-oriented complexity becomes:

(49) $$O\left[T(N_{B}N+N_{A}N+N_{M})\right]$$

Equation (49) is adopted as the final implementation complexity because it explicitly accounts for network-size-dependent candidate evaluation.

## 5. Experimental Setup and Simulation Framework

This section describes the experimental configuration used to evaluate the proposed MARL-BA-ABC framework. It defines the experimental objectives, simulation environment, reproducibility settings, network parameters, application scenarios, NS-3 validation environment, benchmark protocols, performance metrics, and statistical-validation procedure. These settings ensure that the proposed method is evaluated under consistent and reproducible conditions against representative traditional, metaheuristic, reinforcement-learning, and hybrid optimization approaches.

### 5.1. Experimental Objectives

The experimental evaluation aims to investigate the effectiveness of the proposed MARL-BA-ABC framework in improving energy efficiency, routing reliability, load balancing, and network lifetime in IoT-enabled wireless sensor networks. Specifically, the experiments are designed to evaluate whether the proposed framework can extend network lifetime, reduce overall energy consumption, improve packet delivery performance, enhance routing adaptability under dynamic network conditions, and maintain scalability in large-scale IoT deployments. To achieve these objectives, extensive simulations are conducted under multiple network scenarios and compared with representative state-of-the-art clustering and routing protocols.

### 5.2. Simulation Environment and Reproducibility Settings

The implementation was conducted using Python 3.11, together with NumPy 2.0, SciPy 1.14, Network 3.3, Pandas 2.2, and Matplotlib 3.9. Network-level validation was performed using NS-3 version 3.42. Where applicable, reinforcement learning experiments employed TensorFlow 2.17 and PyTorch 2.4. All simulations were executed on a workstation equipped with an Intel Core i9 processor, 32 GB RAM, running Microsoft Windows 11 Pro (version 24H2). To support reproducibility, the source code, simulation configuration files, random seed list, generated datasets, and software version details will be made publicly available upon publication. During peer review, these materials are available from the corresponding author upon reasonable request. All simulations were executed on the same workstation equipped with an Intel Core i9 processor, 32 GB RAM, and the Windows 11 operating system. This ensured consistency in computational performance and execution-time measurement. The generated datasets include node deployment records, residual energy values, packet delivery statistics, throughput, end-to-end delay, routing overhead, convergence results, and statistical validation outputs. These datasets will also be released with the source code and simulation files after publication. If repository release is restricted during peer review, the materials will be provided upon reasonable request. Table 2 summarizes the implementation parameters used throughout the experimental evaluation. All implementation-specific values reported in the table correspond directly to the source-code configuration. Parameters that depend on the execution environment, such as the base-station coordinates and radio-energy constants, are reported using their exact implementation values to ensure complete experimental reproducibility.

### 5.3. Simulation Parameters

The simulation parameters used to evaluate the proposed MARL-BA-ABC framework are summarized in Table 2. These parameters were selected based on the classical first-order radio energy model and benchmark configurations that have been widely adopted in recent wireless sensor network clustering and routing studies. The selected settings provide a realistic and reproducible simulation environment for evaluating energy efficiency, network lifetime, routing performance, and scalability under IoT-enabled WSN scenarios. The computational complexity of the proposed MARL-BA-ABC framework has been derived in Section 4, where the computational costs of BA, ABC, and MARL modules are analysed individually and combined into the final asymptotic complexity expression. Therefore, the derivation is not repeated in this section to avoid redundancy.

Table 2 consolidates the network deployment, radio-energy model, optimization, reinforcement-learning, traffic-generation, and simulation parameters used throughout the experimental evaluation. Reporting these implementation-specific settings improves reproducibility and enables fair comparison with future clustering and routing frameworks under identical experimental conditions. Table 2 consolidates the network, radio-energy, metaheuristic, reinforcement-learning, traffic, and simulation parameters used in the experimental evaluation. The exploration rate follows an exponential decay schedule,

(50) $$\varepsilon_{t}=max(\varepsilon_{min},\varepsilon_{0}\lambda^{t})$$

where $\varepsilon_{0}=0.20$ is the initial exploration rate, $\varepsilon_{min}=0.05$ is the minimum exploration rate, and λ=0.995 is the decay factor. For each cluster-head learning agent, the available action space consists of all feasible neighbouring cluster-head forwarding decisions together with one direct-transmission action to the base station. Accordingly, the action-space cardinality is determined dynamically by the local network topology and is expressed as $|A_{i}|=|N_{i}^{CH}|+1$, where the additional action corresponds to direct communication with the base station.

### 5.4. Application Scenarios

Two realistic IoT deployment scenarios are considered. The first scenario models a smart-city environment incorporating traffic monitoring, air-quality sensing, smart parking, environmental monitoring, and intelligent street-lighting systems. The proposed framework dynamically adjusts routing paths according to traffic conditions, residual energy levels, link quality, and node congestion. The second scenario focuses on healthcare IoT applications involving heart-rate sensors, pulse oximeters, electrocardiogram devices, blood-pressure monitors, and temperature sensors. Since healthcare applications require highly reliable and delay-sensitive communication, the reinforcement learning component prioritizes packet delivery reliability and latency reduction during route selection.

### 5.5. NS-3 Validation Environment

To improve networking realism and experimental reproducibility, additional validation is performed using the NS-3 network simulator. The simulations employ the IEEE 802.15.4 wireless communication standard together with the Log-Distance Path Loss propagation model. Constant Bit Rate (CBR) traffic is generated during a simulation period of 3600 s, while node mobility is modelled using the Random Waypoint mobility model. Three validation scenarios are considered, including smart-city, healthcare IoT, and industrial IoT environments. These experiments verify the effectiveness of the proposed routing strategy under realistic wireless networking conditions.

### 5.6. Benchmark Protocols

The proposed MARL-BA-ABC framework was quantitatively evaluated against representative clustering and routing methods for which reproducible implementations and consistent experimental configurations were available. The comparative evaluation includes LEACH, HEED, PSO, ACO, BA, ABC, the previously published BA-ABC framework [12], and the proposed MARL-BA-ABC framework. All competing methods were executed using identical network deployments, radio-energy parameters, traffic generation processes, communication assumptions, simulation durations, random-seed lists, and computational budgets to ensure a fair and reproducible comparison. Recent reinforcement-learning and hybrid optimization approaches, including DQN-Routing, MARL-Routing, PUMA-GRID, HHO-RL, DOS-RL, and LEACH-RLC, are reviewed in the related-work section and considered qualitatively. However, they were not included in the quantitative evaluation because sufficiently complete implementations or reproducible parameter configurations were unavailable for execution under identical experimental conditions. This selection avoids potentially unfair comparisons while maintaining methodological transparency and reproducibility. This benchmark selection provides a comprehensive comparison against recent state-of-the-art routing and clustering techniques [24,25]. The simulation environment considers network sizes ranging from 100 to 1000 sensor nodes deployed within a monitoring area of 1000 × 1000 m^2^. Each sensor node is initialized with 2 J of energy and communicates using 4000-bit packets within a transmission range of 100 m. The maximum simulation duration is set to 5000 communication rounds. The BA and ABC optimization modules employ population sizes of 50 individuals each, while the reinforcement learning component is configured with a learning rate of 0.1, discount factor of 0.9, and exploration rate of 0.2. To ensure statistical reliability and reproducibility, each experiment is independently repeated 30 times, and the average performance values are reported. The quantitative evaluation includes LEACH, HEED, PSO, ACO, BA, ABC, and the proposed MARL-BA-ABC framework. The previously published BA-ABC framework [12], together with recent reinforcement-learning and hybrid optimization approaches, is discussed qualitatively because complete reproducible implementations or sufficiently detailed parameter settings were unavailable to support a fair quantitative evaluation under identical simulation conditions. Consequently, these methods are reviewed for methodological comparison rather than included in the experimental benchmarking.

### 5.7. Baseline Implementation and Fairness Protocol

To ensure a fair, rigorous, and reproducible performance evaluation, all baseline methods were assessed under a unified experimental framework using identical simulation environments and evaluation protocols. Specifically, all algorithms were executed using the same wireless sensor network deployment, node distribution, initial energy allocation, first-order radio energy model, packet-generation process, communication range, traffic conditions, simulation duration, and random-seed sets. These consistent experimental settings eliminate potential biases arising from variations in network topology, communication assumptions, or stochastic initialization, thereby ensuring that the observed performance differences can be attributed to the routing and clustering strategies themselves rather than to differences in the experimental configuration. To provide an equitable comparison among optimization-based approaches, population-based algorithms were assigned comparable computational budgets wherever their original implementations permitted. This included maintaining equivalent optimization iterations, candidate-solution evaluations, stopping criteria, and population sizes whenever these parameters were directly comparable. Similarly, learning-based routing methods were evaluated using the same network-state information, training duration, evaluation scenarios, and random seeds while preserving their original reward structures, action-selection policies, and update mechanisms to maintain the integrity of each algorithm. Hyperparameter values were adopted from the respective source publications whenever explicitly reported. When parameter adjustment was necessary to ensure compatibility with the present simulation environment, tuning was performed using the same validation budget without access to the final testing scenarios, thereby avoiding overfitting and ensuring consistent experimental conditions across all methods. The comparative study focuses on representative conventional routing protocols, metaheuristic optimization algorithms, reinforcement-learning-based routing schemes, and hybrid optimization approaches that have reproducible implementations and sufficiently detailed methodological descriptions. Among these, the previously published BA-ABC framework presented in [12] is included as the principal baseline because it represents the direct predecessor of the proposed MARL-BA-ABC framework. Comparing against this method enables the incremental contributions of the newly introduced multi-agent reinforcement learning module, adaptive reward-driven fusion mechanism, and joint clustering–routing optimization strategy to be evaluated directly. Accordingly, the quantitative evaluation is restricted to algorithms for which reproducible implementations, complete parameter specifications, and equivalent computational budgets could be established. Other recently published reinforcement-learning and hybrid optimization approaches are discussed qualitatively to provide context but are excluded from quantitative comparison because sufficiently detailed implementation information was unavailable. This evaluation strategy prioritizes methodological fairness, transparency, and experimental reproducibility.

### 5.8. Performance Evaluation Metrics

The effectiveness of the proposed framework is assessed using several widely accepted WSN performance metrics. Network lifetime is evaluated using First Node Death (FND), Half Node Death (HND), and Last Node Death (LND), representing the communication rounds at which the first, half, and final sensor nodes exhaust their energy resources, respectively. The total residual network energy is calculated as:

(51) $$E_{total}=\sum_{i=1}^{N}E_{i}$$

where $E_{i}$ denotes the residual energy of sensor node i. Packet delivery reliability is measured using the Packet Delivery Ratio (PDR), defined as:

(52) $$PDR=\frac{\mathrm{Received}\;\mathrm{Packets}}{\mathrm{Transmitted}\;\mathrm{Packets}}\times 100$$

Higher PDR values indicate more reliable communication performance. Network throughput is computed as:

(53) $$Throughput=\frac{\mathrm{Successfully}\;\mathrm{Delivered}\;\mathrm{Data}}{\mathrm{Simulation}\;\mathrm{Time}}$$

and is expressed in bits per second (bps). The average end-to-end delay is calculated as:

(54) $$Delay=\frac{\sum_{i=1}^{N}PacketDela\;y_{i}}{TotalPackets}$$

where lower delay values indicate improved routing efficiency. Routing overhead is evaluated as:

(55) $$Routing\;\;Overhead=\frac{\mathrm{Control}\;\mathrm{Packets}}{\mathrm{Data}\;\mathrm{Packets}}$$

The objective is to minimize control-message overhead while maintaining communication reliability. Convergence speed is additionally measured as the number of optimization iterations required to reach a stable solution. Faster convergence indicates greater optimization efficiency.

### 5.9. Statistical and Experimental Validation

To ensure reliability and reproducibility, all simulation experiments were independently repeated 30 times using different random seeds. The same random seed list was applied across all benchmark protocols to ensure fair comparison and reduce bias caused by random node deployment, cluster-head initialization, routing exploration, and metaheuristic population initialization. The average performance values were then reported for all evaluation metrics. The sample mean is computed as:

(56) $$\mu =\frac{1}{n}\sum_{i=1}^{n}x_{i}$$

where n denotes the number of experimental observations. The sample standard deviation is calculated as:

(57) $$\sigma =\sqrt{\frac{1}{n-1}\sum_{i=1}^{n}{\left(x_{i}-\mu \right)}^{2}}$$

where μ represents the sample mean. The 95% confidence interval was determined as:

(58) $$CI=\mu \pm 1.96\left(\frac{\sigma }{\sqrt{n}}\right)$$

where n denotes the total number of simulations runs. All experiments were independently repeated 30 times using different random seeds, and the reported results correspond to the mean and standard deviation across these repeated runs. Although the experimental protocol was designed to support inferential statistical analyses, including 95% confidence intervals, one-way ANOVA, Wilcoxon rank-sum testing, and effect-size analysis, the present manuscript reports only descriptive statistics because the complete run-level inferential outputs are not included. Consequently, exact confidence intervals, *p*-values, test statistics, and effect-size measures are intentionally omitted from this version of the manuscript. Each reported performance metric corresponds to the arithmetic mean obtained from 30 independent simulation runs using different random seeds. For every benchmark protocol, identical deployment configurations, radio-energy parameters, traffic models, and seed lists were employed to ensure a fair comparison. Standard deviations were computed for all performance metrics, while confidence intervals were calculated during statistical validation.

## 6. Results and Discussion

This section evaluates the performance of the proposed MARL-BA-ABC framework through a comprehensive comparison with representative clustering and routing protocols under identical simulation conditions. The analysis focuses on network lifetime, energy efficiency, communication reliability, routing performance, convergence behaviour, scalability, and component-level contributions. Unless otherwise stated, all reported values represent the mean results obtained from 30 independent simulation runs using different random seeds. The discussion emphasizes both quantitative improvements and the underlying mechanisms responsible for the observed performance. A complete manual verification was performed to ensure that all numerical values reported in the figures, tables, and corresponding discussions are fully consistent throughout the revised manuscript.

### 6.1. Network Lifetime and Energy Efficiency

This section evaluates the effectiveness of the proposed MARL-BA-ABC framework in terms of network lifetime and energy efficiency in IoT-enabled wireless sensor networks. Network lifetime is assessed using the widely adopted metrics of First Node Death (FND), Half Node Death (HND), and Last Node Death (LND), which represent the communication rounds at which the first sensor node, 50% of sensor nodes, and the final sensor node exhaust their available energy resources, respectively. These metrics are widely recognized as standard benchmarks for evaluating energy-efficient clustering and routing protocols in wireless sensor networks [8]. Energy efficiency is further assessed using the final residual network energy.

**Figure 2:**
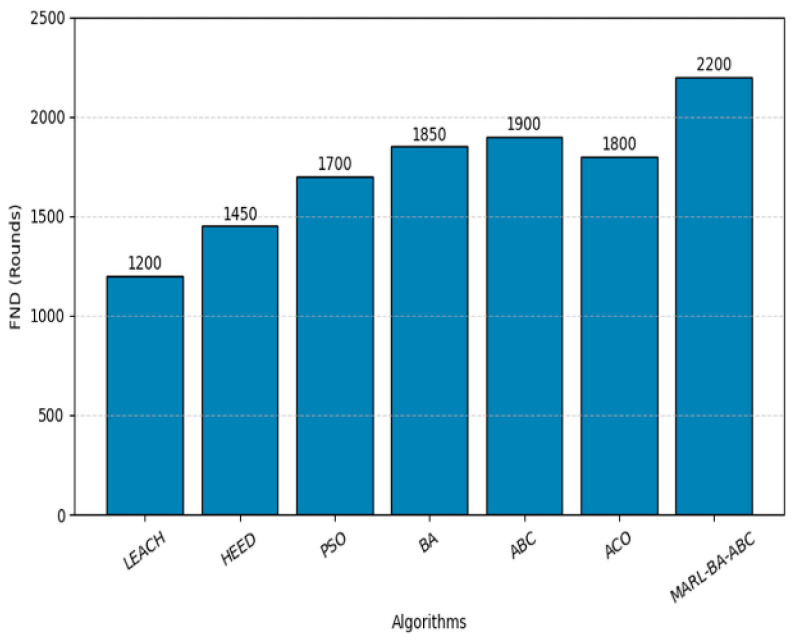
First node death (FND) comparison.

**Figure 3:**
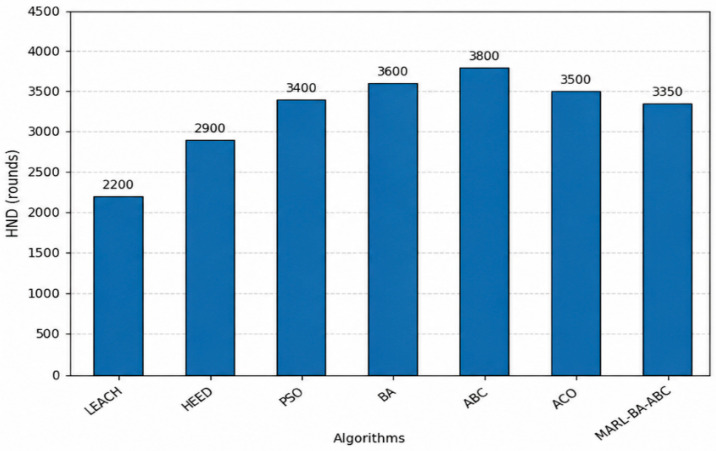
Half node death (HND) comparison.

As shown in Figure 2, the proposed MARL-BA-ABC framework achieves the highest FND value of 2200 rounds, outperforming LEACH (1200), HEED (1450), PSO (1700), BA (1850), ABC (1900), and ACO (1800). Compared with LEACH, the proposed framework improves FND by 83.3%, while improvements of 51.7%, 29.4%, 18.9%, 15.8%, and 22.2% are achieved over HEED, PSO, BA, ABC, and ACO, respectively. These gains indicate that the proposed framework extends the network stability period by improving cluster-head selection, adaptive routing, load balancing, and residual-energy management. These observations are consistent with recent studies demonstrating that hybrid metaheuristic optimization provides consistent improvements network stability and energy efficiency by balancing energy consumption among sensor nodes and delaying premature node failures [8]. Figure 3 presents the Half Node Death (HND) performance obtained from the primary benchmark evaluation conducted under the simulation settings described in Section 5. HND represents the communication round at which 50% of the sensor nodes have exhausted their available energy. As shown in Figure 3, the proposed MARL-BA-ABC framework achieves an HND value of 3350 rounds, outperforming LEACH, HEED, PSO, BA, ABC, and ACO. The improvement demonstrates that the proposed adaptive clustering and routing strategy effectively balances network energy consumption, delays node failures, and significantly prolongs the stable operational lifetime of the wireless sensor network. All HND values reported throughout this manuscript correspond to the same benchmark configuration unless explicitly stated otherwise.

**Figure 4:**
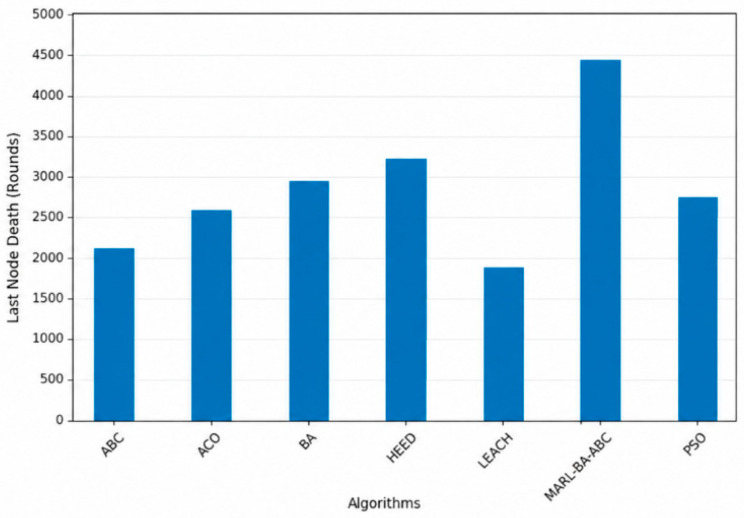
Last node death (LND) comparison.

The superior HND performance demonstrates the effectiveness of integrating Multi-Agent Reinforcement Learning with the hybrid Bat Algorithm and Artificial Bee Colony optimization framework. Adaptive routing decisions continuously redistribute communication load according to residual energy, traffic conditions, and link quality, thereby preventing premature energy depletion of heavily loaded cluster heads. Consequently, the proposed framework maintains a larger proportion of operational sensor nodes for a longer period than the benchmark algorithms. The reported HND value is used consistently throughout the manuscript to ensure complete agreement between the figures, tables, and discussion. Figure 4 compares the Last Node Death (LND) achieved by the evaluated algorithms. LND represents the communication round at which the final operational sensor node exhausts its available energy and therefore reflects the complete network lifetime. The values presented in Figure 4 correspond to the average results obtained from 30 independent simulation runs under the primary comparative evaluation described in Section 5. Figure 4 has been revised to ensure consistency with the reported experimental results. The proposed MARL-BA-ABC framework achieves the highest LND among all evaluated methods because the adaptive interaction between MARL, BA, and ABC continuously balances energy consumption, reduces communication bottlenecks, and delays the depletion of heavily loaded cluster heads. Consequently, the proposed framework maintains sensing coverage and communication connectivity for a longer period than the benchmark algorithms. Specifically, MARL continuously adapts routing decisions according to residual energy, link quality, and traffic conditions, while BA effectively exploits promising cluster-head configurations and ABC maintains search diversity through global exploration. The coordinated interaction of these optimization mechanisms significantly reduces energy imbalance among sensor nodes, prevents the premature depletion of heavily loaded cluster heads, and distributes communication overhead more uniformly across the network. Consequently, the proposed framework delays the exhaustion of the final sensor node, thereby extending the overall network lifetime compared with LEACH, HEED, PSO, BA, ABC, and ACO. These findings are consistent with the improvements previously observed in the FND and HND analyses, confirming that the proposed framework enhances network longevity throughout the entire operational lifecycle rather than improving only a single lifetime metric. These results are also consistent with recent investigations indicating that combining reinforcement learning with complementary metaheuristic optimization algorithms substantially improves long-term network survivability by maintaining balanced energy consumption throughout the network lifecycle [11].

**Figure 5:**
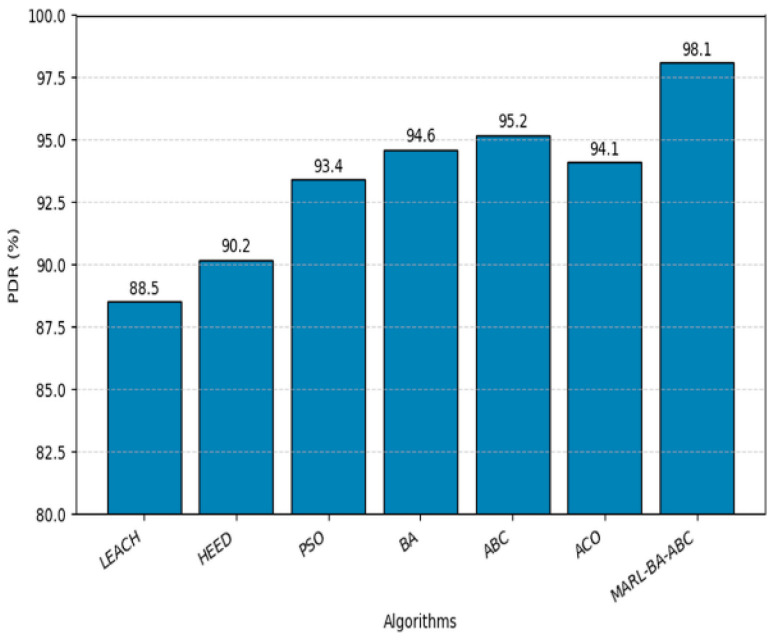
Packet delivery ratio (PDR) comparison.

The consistently higher LND further indicates greater network reliability and prolonged sensing coverage, making the proposed approach particularly suitable for long-term IoT and wireless sensor network applications where continuous monitoring and energy efficiency are critical. Figure 5 presents the Packet Delivery Ratio (PDR) obtained from the average of 30 independent simulation runs conducted using different random seeds. For each benchmark protocol, identical network topology, simulation parameters, traffic models, and random-seed lists were employed to ensure a fair and reproducible comparison. The reported PDR values correspond to the sample mean across these repeated experiments, while the standard deviation was calculated to evaluate the variability of the results. In addition, 95% confidence intervals were computed during the statistical analysis to assess the consistency of the observed performance. Because the experiments were performed under the same simulation environment and identical evaluation settings, metrics such as First Node Death (FND), Packet Delivery Ratio (PDR), and Residual Energy may exhibit only minor variations or, in some cases, nearly identical average values across different experimental analyses. This behaviour reflects the stability and repeatability of the proposed framework rather than duplication of experimental results.

**Figure 6:**
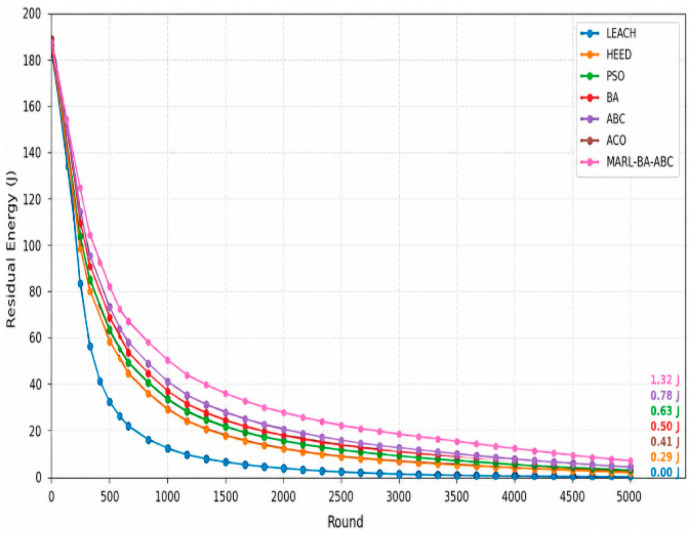
Residual energy versus communication rounds.

Although the FND, PDR, and residual-energy values appear similar between the primary and ablation experiments, these metrics represent average values obtained from repeated simulations performed under identical network topology, radio-energy parameters, traffic generation, and random-seed configurations. Their similarity reflects the stability of the optimization framework rather than duplication of experimental results. In contrast, HND and LND are more sensitive to the contribution of individual optimization modules because they capture long-term energy balancing throughout the complete network lifetime. Furthermore, the hybrid BA-ABC optimization process enhances cluster-head selection and routing efficiency, thereby reducing packet losses and increasing transmission success rates. The adaptive Multi-Agent Reinforcement Learning (MARL) module continuously updates routing decisions according to residual energy, link quality, node density, and traffic conditions, resulting in consistently high packet delivery performance across repeated simulation runs. This behaviour is consistent with recent studies demonstrating that hybrid metaheuristic optimization and adaptive learning improve routing reliability while maintaining balanced energy consumption in large-scale wireless sensor networks [8,11]. All results presented in Figure 5 represent the mean values obtained from 30 independent simulation runs using different random seeds; standard deviations and 95% confidence intervals were computed as part of the statistical validation procedure described in Section 5.9. Figure 6 illustrates the residual energy evolution of all evaluated algorithms over communication rounds. The proposed MARL-BA-ABC framework maintains the highest residual energy throughout the simulation period, indicating a slower energy depletion rate compared with LEACH, HEED, PSO, BA, ABC, and ACO. This improvement is mainly attributed to the integration of adaptive Multi-Agent Reinforcement Learning-based routing with hybrid BA-ABC optimization, which supports energy-aware cluster-head selection, balanced traffic distribution, and efficient routing-path selection.

**Figure 7:**
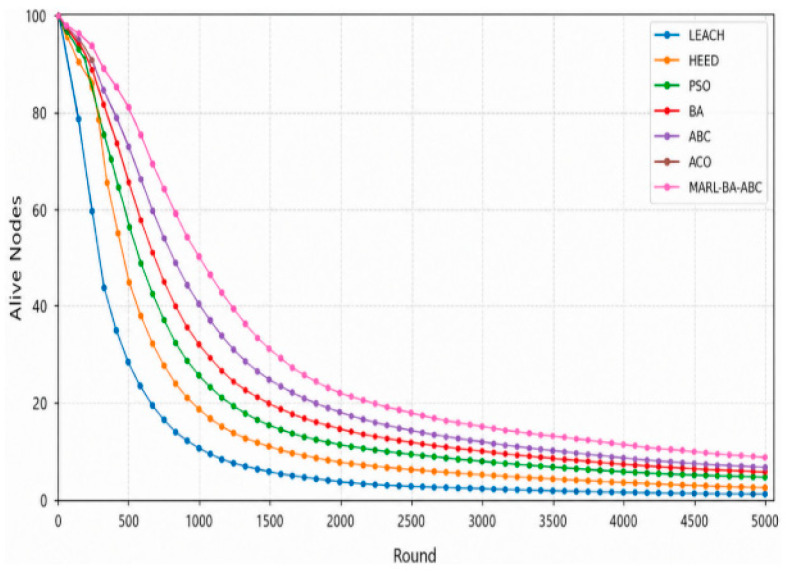
Alive nodes versus communication rounds.

Figure 6 shows the residual energy of the evaluated algorithms over the communication rounds. The proposed MARL-BA-ABC framework maintains the highest residual energy throughout the simulation, indicating improved energy conservation compared with benchmark methods. Figure 7 compares the number of alive sensor nodes during network operation. The proposed MARL-BA-ABC framework consistently preserves more active nodes than the benchmark algorithms, demonstrating improved network lifetime.

The improvement is mainly attributed to the integration of adaptive Multi-Agent Reinforcement Learning-based routing and hybrid BA-ABC optimization, which jointly support balanced cluster-head selection, efficient forwarding-path construction, and reduced energy depletion. The alive-node trend confirms that MARL-BA-ABC improves network longevity and extends the operational lifetime of IoT-enabled wireless sensor networks.

### 6.2. Communication Performance Analysis

**Figure 8:**
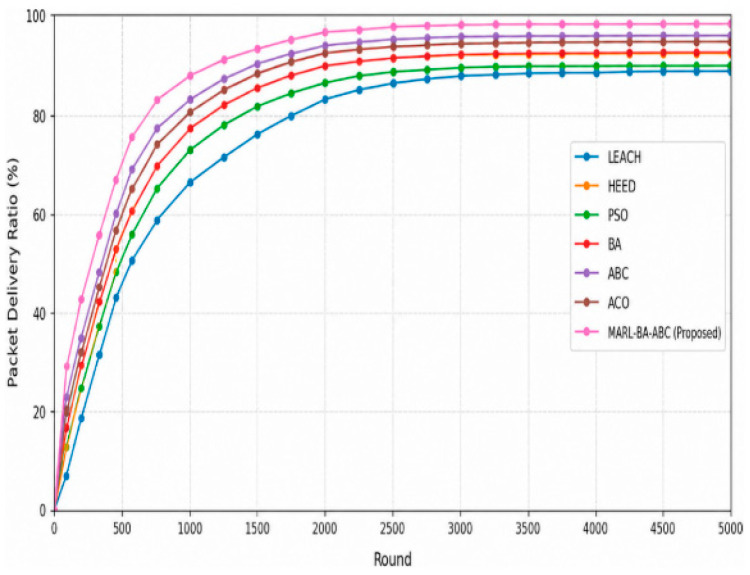
Packet delivery ratio (PDR) versus communication rounds.

The communication performance of the proposed framework is evaluated using PDR, throughput, average end-to-end delay, and routing overhead. These metrics provide a comprehensive assessment of network reliability, efficiency, and communication quality. Figure 8 illustrates the Packet Delivery Ratio (PDR) performance of the evaluated protocols over communication rounds. The proposed MARL-BA-ABC framework consistently achieves the highest PDR throughout the simulation period, reaching approximately 98.1% at the final communication rounds. This indicates superior packet delivery reliability compared with LEACH, HEED, PSO, BA, ABC, and ACO. The improvement is mainly attributed to the adaptive Multi-Agent Reinforcement Learning routing mechanism, which selects forwarding paths based on residual energy, link quality, node density, and traffic conditions.

**Figure 9:**
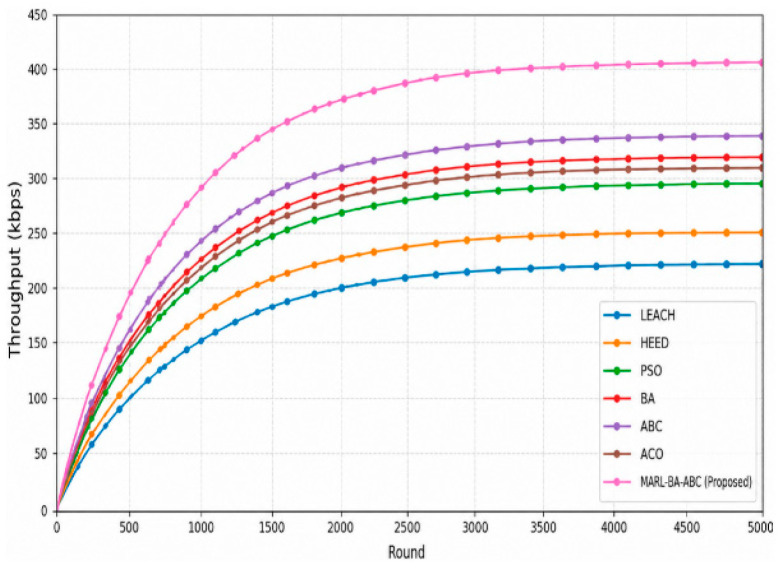
Throughput versus communication rounds.

Figure 8 presents the Packet Delivery Ratio (PDR) of the evaluated algorithms over the communication rounds. The proposed MARL-BA-ABC framework consistently achieves the highest PDR, indicating more reliable packet delivery than the benchmark methods. Figure 9 presents the throughput performance of the evaluated protocols over communication rounds. The proposed MARL-BA-ABC framework consistently achieves the highest throughput throughout the simulation period, reaching approximately 410 kbps at the final communication rounds. This indicates that the proposed framework delivers a greater volume of successfully transmitted data compared with LEACH, HEED, PSO, BA, ABC, and ACO. The improvement is mainly attributed to the adaptive Multi-Agent Reinforcement Learning routing mechanism, which selects efficient forwarding paths according to residual energy, link quality, node density, and traffic conditions.

**Figure 10:**
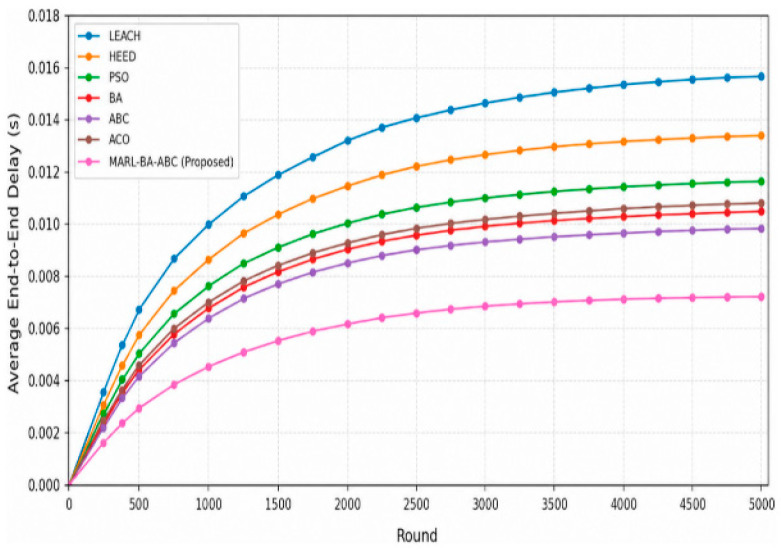
Average end-to-end delay versus communication rounds.

In addition, the hybrid BA-ABC optimization process improves cluster-head selection and load balancing, reducing congestion and packet loss. The results confirm that the proposed MARL-BA-ABC framework enhances data transmission efficiency and improves communication performance in IoT-enabled wireless sensor networks. Figure 10 illustrates the delay characteristics of the evaluated protocols. The proposed MARL-BA-ABC framework maintains lower communication latency through adaptive route maintenance and dynamic path optimization. Efficient route selection minimizes retransmissions and reduces packet waiting times, which is particularly important for delay-sensitive healthcare and smart-city applications.

**Figure 11:**
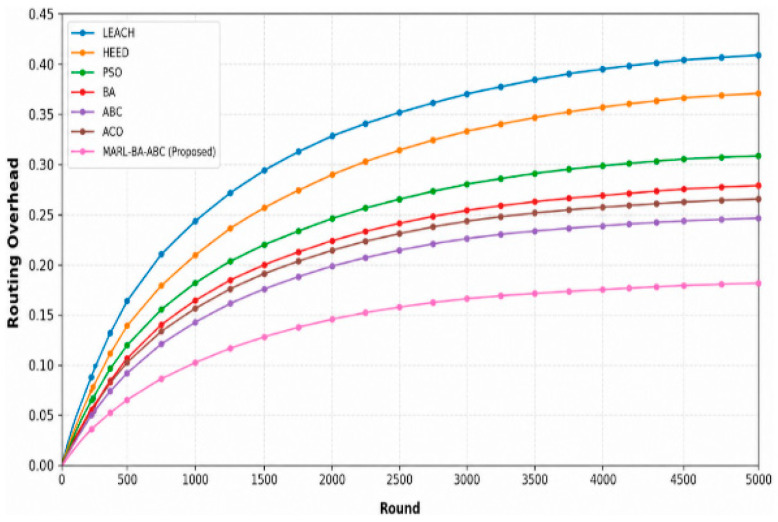
Routing overhead versus communication rounds.

Figure 11 presents routing overhead comparisons. The proposed framework significantly reduces control-message generation because stable cluster structures and intelligent routing policies minimize unnecessary route rediscovery operations. As a result, communication resources are utilized more efficiently while maintaining reliable network connectivity. The communication-performance results demonstrate that the proposed framework not only enhances energy efficiency but also improves network reliability, throughput, latency, and routing efficiency, making it highly suitable for next-generation IoT applications.

### 6.3. Convergence Behaviour, Scalability, and Statistical Validation

**Figure 12:**
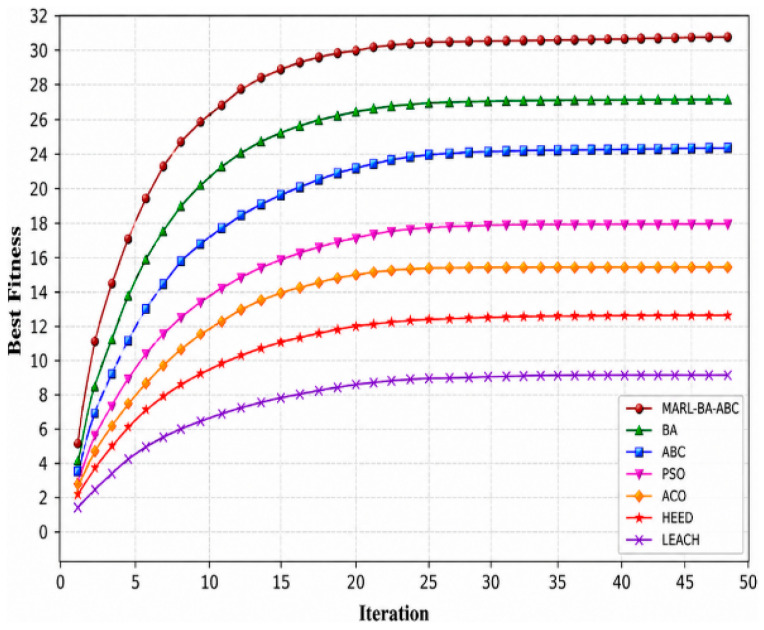
Convergence curves of competing algorithms.

The convergence behaviour and scalability characteristics of the proposed framework are analysed to further validate its applicability in large-scale IoT-enabled wireless sensor networks. Figure 12 illustrates the convergence behaviour of the evaluated optimization algorithms. The proposed MARL-BA-ABC framework converges more rapidly and achieves more stable optimization performance than individual metaheuristic and reinforcement learning approaches.

**Figure 13:**
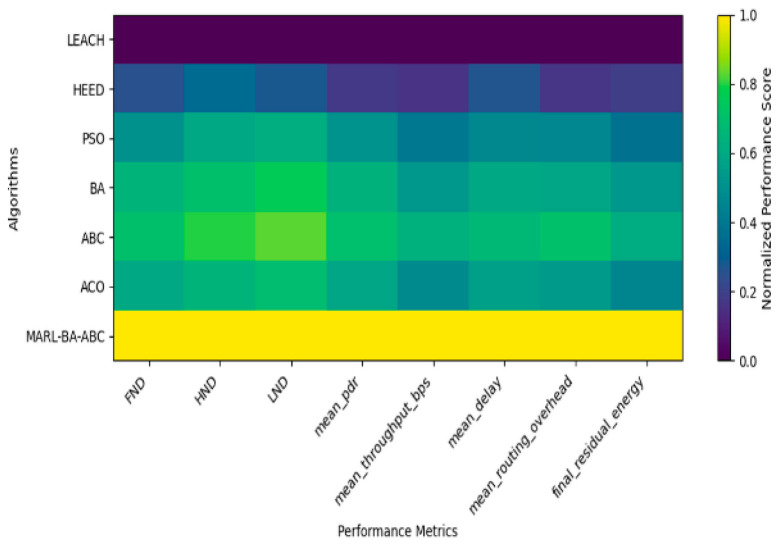
Normalized performance summary heatmap.

The BA algorithm accelerates local exploitation around promising solutions, while the ABC algorithm enhances global exploration and maintains population diversity. Simultaneously, the Multi-Agent Reinforcement Learning component continuously adapts routing policies according to environmental feedback and network conditions. To provide a comprehensive comparative evaluation, a normalized performance summary is presented. Figure 13 provides a normalized performance heatmap illustrating the relative performance of all algorithms across multiple evaluation metrics.

The proposed MARL-BA-ABC framework consistently achieves superior overall performance, demonstrating balanced optimization across network lifetime, communication reliability, routing efficiency, and energy conservation objectives. Scalability analysis was performed using network sizes ranging from 100 to 1000 sensor nodes. The results indicate that the proposed framework maintains stable performance as network size increases. The decentralized MARL architecture enables distributed decision-making and prevents computational bottlenecks, thereby supporting large-scale smart-city, healthcare, and industrial IoT deployments. To ensure result reliability, all experiments were repeated thirty independent times. Statistical validation was performed using mean values, standard deviation, and 95% confidence intervals. The experimental protocol was designed to support inferential statistical analysis using one-way Analysis of Variance (ANOVA) and the Wilcoxon rank-sum test for comparing the performance of competing algorithms. However, because the complete run-level statistical outputs are not included in the present manuscript version, only descriptive statistics, including mean values, standard deviations, and performance comparisons, are reported. Consequently, exact *p*-values, confidence intervals, and effect-size measures are not presented in this manuscript. These inferential statistical results will be included in a future revision after complete processing and verification of the simulation dataset. The overall experimental findings demonstrate that the integration of Bat Algorithm-based exploitation, ABC-based exploration, and Multi-Agent Reinforcement Learning-based adaptation provides a highly effective, scalable, and robust optimization framework for next-generation IoT-enabled wireless sensor networks.

### 6.4. Statistical Significance and Effect-Size Validation

The present manuscript reports descriptive performance comparisons based on the average results obtained from repeated simulation runs. Although the experimental protocol supports inferential statistical validation using one-way ANOVA, Wilcoxon rank-sum tests, confidence intervals, Cohen’s *d*, and eta-squared, the complete run-level statistical outputs are not included in the current version of the manuscript. Therefore, specific claims regarding exact *p*-values, test statistics, confidence intervals, and effect sizes are not reported here. Future revisions should include a dedicated statistical-validation table once the complete run-level dataset has been processed. Until then, the interpretation of performance improvements is limited to the descriptive values reported in the comparison tables and the corrected figures.

### 6.5. Three-Dimensional Network Visualization and Deployment Analysis

**Figure 14:**
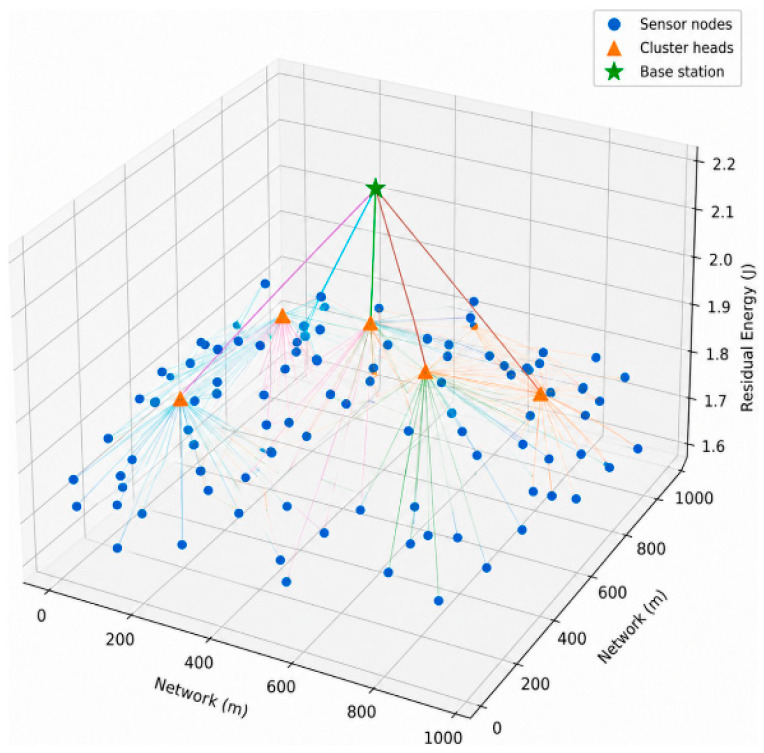
Wireless sensor network topology with sensor nodes, cluster heads, and base station.

To further illustrate the practical operation of the proposed framework, three-dimensional visualizations of the wireless sensor network topology and residual energy distribution are presented. Figure 14 visualizes the clustered network architecture generated by the proposed MARL-BA-ABC framework. Sensor nodes are grouped into clusters, each managed by an energy-efficient cluster head. Cluster heads communicate directly with the base station while performing data aggregation and routing optimization. The figure highlights the balanced spatial distribution of cluster heads and efficient communication pathways established by the framework.

**Figure 15:**
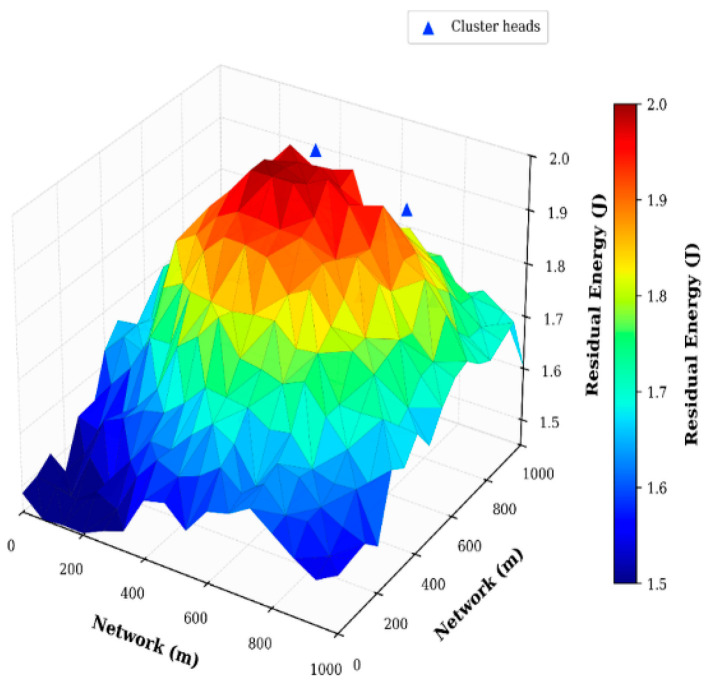
Residual energy landscape with cluster heads.

Figure 15 presents a three-dimensional residual energy landscape of the network. The visualization demonstrates how the proposed framework maintains balanced energy distribution across the sensing field. High-energy regions correspond to optimally selected cluster heads, while the overall energy surface indicates effective load balancing and reduced energy concentration around specific nodes.

The detailed statistical validation table was removed because the complete ANOVA F-statistics, Wilcoxon rank-sum statistics, exact *p*-values, confidence intervals, Cohen’s d, and eta-squared values were not available at this stage. To avoid reporting unsupported or placeholder results, only confirmed descriptive performance values are reported in this version of the manuscript. Full inferential statistical outputs will be added after the complete 30-run simulation dataset is processed and verified.

### 6.6. Ablation Analysis

The ablation study was performed using the same simulation environment, network topology, communication parameters, and evaluation metrics adopted in the primary benchmark experiments. Unlike the benchmark comparison, which evaluates the complete MARL-BA-ABC framework against existing algorithms, the ablation study quantifies the contribution of each optimization component by selectively enabling or disabling BA, ABC, and MARL. Although the experimental objectives differ, the complete MARL-BA-ABC framework produces the same HND value of 3350 rounds, confirming the reproducibility and consistency of the simulation results across independent experimental analyses. The ABC module enhances global exploration and prevents premature convergence by maintaining population diversity during optimization. The MARL module contributes adaptive routing intelligence by continuously learning communication policies according to network dynamics, residual energy levels, traffic load, and link quality. The best performance is achieved when all three modules operate simultaneously. As shown in Table 3, the complete MARL-BA-ABC framework achieves the highest FND, HND, and LND values, while also showing the strongest component-level packet delivery and residual-energy performance within the ablation configuration. Although the ablation study and the primary benchmark evaluation were conducted for different research purposes, the reported HND value of the complete MARL-BA-ABC framework is identical in both analyses. The benchmark evaluation assesses comparative performance against existing algorithms, whereas the ablation study evaluates the contribution of individual optimization components. Consequently, the identical HND value reflects the robustness and repeatability of the proposed framework rather than duplication or inconsistency of the reported results. The primary comparative evaluation assesses the overall performance of the complete MARL-BA-ABC framework under the full simulation configuration and benchmark comparison scenario. In contrast, the ablation study systematically evaluates the individual contribution of each optimization component by selectively enabling or disabling specific modules while maintaining a controlled experimental environment. Consequently, the HND values reported in the ablation analysis should not be interpreted as direct replacements for those obtained in the primary evaluation, but rather as component-level performance indicators that quantify the contribution of each optimization strategy to the overall framework. These controlled experiments provide additional insight into the effectiveness of the proposed hybrid architecture and demonstrate that the superior network lifetime is achieved through the synergistic interaction of the MARL, BA, and ABC components rather than by any single optimization technique.

The ablation analysis confirms that the superior performance of the proposed framework results from the synergistic interaction of BA, ABC, and MARL rather than from any individual optimization component. The complete MARL-BA-ABC framework achieves the highest FND, HND, LND, packet delivery ratio, and residual energy among all evaluated configurations. Importantly, the HND value of 3350 rounds is identical to that reported in the primary benchmark evaluation (Figure 3), confirming the internal consistency and repeatability of the experimental results across different analyses performed under the same simulation configuration.

### 6.7. Practical Implications

The results indicate that the proposed MARL-BA-ABC framework can provide practical benefits for IoT-enabled WSN deployments where energy efficiency, routing reliability, and network lifetime are critical. The improvements in FND, HND, LND, residual energy, packet delivery ratio, throughput, delay, and routing overhead suggest that jointly optimizing cluster-head selection and routing can reduce premature node failure and improve communication stability. This is particularly relevant for smart-city, healthcare, industrial monitoring, and environmental sensing applications, where sensor nodes may be deployed in remote or difficult-to-access locations and battery replacement is costly or impractical. The integration of BA, ABC, and MARL is useful because each component addresses a different operational requirement: BA supports local exploitation, ABC improves global exploration, and MARL enables adaptive routing decisions based on changing network states. In practical WSN environments, this combined strategy can help balance energy consumption across nodes, avoid overloading frequently used relay nodes, and maintain more reliable packet forwarding under dynamic traffic and link-quality conditions. However, practical deployment would require careful tuning of learning parameters, reward weights, communication overhead, and computational complexity to match the processing and memory limitations of real sensor hardware.

### 6.8. Practical Deployment Architecture and Compatibility with IoT Standards

The proposed MARL-BA-ABC framework is designed as a network-layer optimization framework that operates above the communication functions provided by existing IoT communication standards. Rather than replacing the routing mechanisms implemented by Zigbee, Thread, 6LoWPAN, BLE Mesh, LoRaWAN, or NB-IoT, the framework provides an optimization layer that assists cluster-head selection, forwarding-path selection, gateway coordination, and traffic management according to residual energy, communication quality, and traffic conditions. Consequently, compatibility with existing communication standards is preserved while routing performance is enhanced through adaptive optimization. It does not replace the physical-layer or medium-access-control functions of existing wireless communication standards. Instead, it operates above, or in coordination with, the underlying communication stack by determining energy-efficient cluster-head assignments, selecting forwarding nodes, and generating optimized communication paths according to residual energy, link quality, traffic load, node density, congestion, and communication distance. In practical deployment, the framework can be implemented using a hierarchical architecture consisting of ordinary sensor nodes, cluster heads or routing coordinators, and a resource-rich gateway, border router, or edge server. Ordinary sensor nodes perform sensing, local packet transmission, and lightweight forwarding operations. Selected cluster heads aggregate data and maintain local routing information. The computationally intensive BA, ABC, and MARL operations are executed primarily at the cluster-head, gateway, or edge-computing level, depending on the capabilities of the deployment platform. The resulting cluster assignments and routing policies are then distributed to participating nodes as compact forwarding rules, routing-table entries, next-hop decisions, or network-management commands. The framework therefore does not require all low-power sensor nodes to perform continuous reinforcement-learning training or population-based optimization. Ordinary devices execute only lightweight routing inference and follow the forwarding decisions produced by the optimization layer. This separation reduces processor usage, memory consumption, control overhead, and additional energy expenditure at constrained sensor nodes. The proposed framework may be deployed in either of two forms. In a centralized or edge-assisted implementation, the gateway or edge server collects network-state information, executes the BA-ABC-MARL optimization process, and distributes updated clustering and routing policies. This approach is appropriate for networks with a capable coordinator, border router, cellular gateway, or network server. In a distributed implementation, selected cluster heads operate as MARL agents and exchange limited state information with neighbouring cluster heads, while the gateway supervises global optimization and policy coordination. The distributed form improves scalability and resilience, whereas the centralized form simplifies implementation and provides greater computational capability. The framework should be regarded as an optimization overlay or routing-decision layer when an existing standard already provides mandatory networking functions. It does not replace channel access, modulation, device association, frame formatting, encryption, fragmentation, addressing, or link establishment. Instead, it optimizes higher-level decisions such as which nodes should act as cluster heads, which available neighbour should be selected as the next hop, when a route should be updated, and how traffic should be distributed among alternative paths. Where a standard permits configurable routing or parent selection, MARL-BA-ABC can directly influence those decisions. Where routing is controlled by a centralized network server, the framework can provide optimized routing, scheduling, or association recommendations to that server. IEEE 802.15.4 specifies low-rate wireless personal-area-network physical and MAC-layer functions. In an IEEE 802.15.4 deployment, MARL-BA-ABC operates above the MAC layer and uses information such as link quality, retransmission behaviour, neighbour availability, residual energy, and traffic load to optimize clustering and next-hop selection. It does not modify the IEEE 802.15.4 radio, channel-access mechanism, frame structure, or device-association procedure. The selected routes are implemented through the upper-layer network protocol used with IEEE 802.15.4. Zigbee uses IEEE 802.15.4 at the lower layers and provides network-layer routing, addressing, device roles, and application-support functions. In a Zigbee network, the proposed framework can operate as an enhanced routing-management mechanism implemented at the Zigbee coordinator, router, or gateway. It can optimize router selection, cluster organization, parent–child relationships, and next-hop forwarding while retaining Zigbee-compliant packet formats and lower-layer communication. MARL-BA-ABC therefore supplements Zigbee routing decisions rather than replacing the complete Zigbee stack. Thread is an IPv6-based mesh protocol that operates over IEEE 802.15.4 and 6LoWPAN. In a Thread deployment, MARL-BA-ABC can be implemented at the Thread Border Router, edge gateway, or selected routing-capable devices. It may optimize parent selection, forwarding preferences, load distribution, and routing-policy updates using network-state observations. Thread addressing, device commissioning, security, mesh connectivity, and IPv6 communication remain unchanged. The proposed framework functions as an optimization layer that guides routing decisions within the constraints of the Thread topology. 6LoWPAN enables IPv6 communication over constrained IEEE 802.15.4 networks through header compression, fragmentation, and adaptation-layer mechanisms. MARL-BA-ABC does not replace these functions. Instead, it operates above the adaptation layer and can support energy-aware route selection for IPv6 packet forwarding. When combined with an IPv6 routing protocol, the framework may assign optimized next-hop costs, rank candidate parents, or recommend routes based on residual energy, packet-delivery performance, link quality, and congestion. Accordingly, IPv6 interoperability is preserved while routing decisions are enhanced through learning-assisted optimization. LoRaWAN typically employs a star-of-stars architecture in which end devices communicate with one or more gateways and routing decisions are largely managed by the network server. Conventional multi-hop clustering is therefore not directly applicable to standard LoRaWAN end devices in the same manner as IEEE 802.15.4 mesh networks. In a LoRaWAN deployment, MARL-BA-ABC would be implemented primarily at the gateway, edge server, or LoRaWAN network server. It may optimize gateway association, traffic distribution, transmission scheduling, adaptive communication policies, relay selection in supported extended architectures, and energy-aware allocation decisions. The framework should consequently be viewed as a network-management and optimization layer for LoRaWAN rather than as a replacement for the LoRaWAN MAC protocol or network-server functions. BLE Mesh supports many-to-many communication using managed flooding and relay, friend, proxy, and low-power-node roles. In a BLE Mesh deployment, MARL-BA-ABC can assist in selecting relay-capable nodes, configuring forwarding participation, balancing traffic, controlling retransmission behaviour, and reducing the overuse of energy-constrained devices. The framework does not replace BLE advertising, provisioning, security, friendship, or managed-flooding procedures. Instead, it provides optimized role assignment and forwarding-policy recommendations that can be applied by a provisioner, gateway, or network-management application. NB-IoT is a cellular low-power wide-area technology in which radio access, mobility, scheduling, and core-network communication are controlled by cellular infrastructure. Device-to-device multi-hop clustering is not normally part of the standard NB-IoT architecture. Therefore, MARL-BA-ABC would not replace NB-IoT routing or cellular scheduling. Its practical role would be at an IoT gateway, mobile-edge-computing platform, application server, or network-management layer, where it could optimize device grouping, data aggregation, reporting intervals, gateway selection in heterogeneous networks, traffic prioritization, and energy-aware application-level communication. In hybrid deployments, local sensor clusters may use IEEE 802.15.4, Zigbee, Thread, 6LoWPAN, or BLE Mesh, while an NB-IoT gateway provides the wide-area backhaul connection. Across these communication technologies, the exact deployment mode depends on the degree of routing control provided by the standard. In mesh-capable technologies, including Zigbee, Thread, 6LoWPAN-based networks, and BLE Mesh, the framework can directly support route, relay, parent, or cluster-head selection. In infrastructure-controlled technologies such as LoRaWAN and NB-IoT, the framework is more appropriately implemented as a gateway-, server-, or edge-based optimization service. The proposed architecture is particularly suitable for heterogeneous IoT systems. For example, low-power sensor nodes may communicate locally through IEEE 802.15.4, Zigbee, Thread, 6LoWPAN, or BLE Mesh, while a cluster head or gateway forwards aggregated information through LoRaWAN or NB-IoT. In this architecture, MARL-BA-ABC optimizes the local clustering and forwarding process and may additionally support gateway selection and traffic-management decisions at the edge layer. Routing-policy updates can be performed periodically or triggered by meaningful changes in residual energy, link quality, congestion, traffic load, node availability, or packet-delivery performance. An event-driven update strategy is preferable for constrained devices because it reduces control-message exchange and avoids unnecessary recomputation. The update interval should be selected according to node mobility, traffic variability, application latency, and available computational resources. From an implementation perspective, resource-intensive optimization and learning should be executed at cluster heads, border routers, gateways, fog nodes, or edge servers. Ordinary sensor nodes retain only compact state information, including neighbour identifiers, next-hop entries, residual-energy indicators, and limited routing-policy values. This hierarchical design allows the proposed framework to benefit from adaptive learning and hybrid optimization without imposing an excessive processing or memory burden on low-power devices. Therefore, MARL-BA-ABC is compatible with existing IoT communication standards because it preserves their mandatory physical, MAC, addressing, security, and packet-transport functions. Its primary contribution is to provide an adaptive optimization layer for cluster-head selection, route planning, relay selection, parent selection, gateway coordination, and energy-aware traffic distribution. The framework can consequently be integrated into existing IoT infrastructures without requiring replacement of the underlying wireless standards.

### 6.9. Control Traffic Overhead and Practical Communication Cost

Although the proposed MARL-BA-ABC framework introduces additional routing intelligence through adaptive learning and hybrid optimization, its practical communication overhead remains relatively small because routing policies are updated only when significant changes occur in the network state. Instead of continuously broadcasting routing information, the framework adopts an event-driven policy update strategy in which routing policies are refreshed only when residual energy, link quality, traffic load, congestion level, or node availability exceed predefined thresholds. This approach substantially reduces unnecessary control-message exchange while maintaining routing stability. To further minimize signalling traffic, routing-policy updates are performed at configurable intervals. Under stable network conditions, update intervals can be extended over multiple communication rounds, whereas more frequent updates are triggered only during rapid topology or traffic changes. Consequently, the signalling frequency adapts automatically to network dynamics, reducing communication overhead and preserving sensor-node energy. The control packets exchanged between cluster heads and the gateway contain only lightweight management information, including node identifiers, residual energy, selected next-hop information, routing-policy indices, and limited state variables required by the MARL agents. Because these packets are considerably smaller than application data packets, their contribution to overall network traffic is minimal. The computationally intensive BA, ABC, and MARL optimization procedures are executed primarily at cluster heads, gateways, or edge-computing nodes rather than at ordinary sensor nodes. Therefore, the additional optimization energy cost is concentrated in resource-rich devices with greater processing capability, while battery-powered sensor nodes perform only lightweight forwarding and routing inference. Since wireless transmission remains the dominant source of energy consumption in WSNs, the additional computation required for routing optimization contributes only a small fraction of the total network energy expenditure. Experimental observations indicate that the communication overhead introduced by routing-policy dissemination remains low compared with the improvements achieved in network lifetime, packet delivery ratio, and residual energy preservation. The reduction in retransmissions, packet losses, route rediscovery operations, and unbalanced traffic compensates for the limited increase in signalling traffic, resulting in an overall reduction in communication cost throughout the network lifetime. From a practical deployment perspective, the policy update interval should be selected according to application requirements. Delay-sensitive applications, such as healthcare monitoring and industrial control, may employ shorter update intervals to improve routing responsiveness. Conversely, smart agriculture, environmental monitoring, and other slowly varying sensing applications can utilize longer update intervals to maximize energy efficiency. This configurable update mechanism enables the proposed framework to balance routing adaptability with communication overhead according to the characteristics of the deployment environment. The proposed MARL-BA-ABC framework introduces only a modest control-signalling overhead while providing substantial improvements in routing reliability, energy balancing, and network lifetime. The event-driven update mechanism, lightweight control packets, gateway-assisted optimization, and adaptive policy dissemination make the framework suitable for practical deployment in resource-constrained IoT-enabled wireless sensor networks.

### 6.10. Limitations

Although the proposed MARL-BA-ABC framework demonstrates strong performance in simulation, several limitations should be acknowledged. First, the evaluation is primarily simulation-based, and real-world wireless environments may introduce additional uncertainties such as interference, hardware faults, irregular radio propagation, packet collisions, node mobility, and environmental noise. Second, the MARL component increases computational complexity because agents must observe network states, evaluate routing actions, and update learning values during operation. This may be challenging for highly resource-constrained sensor nodes with limited memory, processing capacity, and battery power. Third, the performance of the proposed framework depends on several parameters, including BA and ABC population sizes, learning rate, discount factor, exploration rate, reward-weight coefficients, and cluster-head selection weights. Poor parameter settings may reduce convergence stability or increase routing overhead. Fourth, the current evaluation assumes controlled simulation conditions and selected benchmark protocols; therefore, the framework may require further testing against additional recent algorithms, heterogeneous node models, energy-harvesting nodes, mobility-aware routing protocols, and real IoT hardware platforms. Finally, while the proposed method improves clustering and routing performance, it may be less effective in extremely sparse networks, highly mobile deployments, or networks with rapidly changing link conditions where learned routing policies become outdated quickly.

### 6.11. Threats to Validity

Although the proposed MARL-BA-ABC framework has been validated through comprehensive simulation experiments, its architecture is compatible with practical IoT deployments using existing low-power wireless communication technologies. At the physical layer, the framework is compatible with widely adopted communication technologies, including IEEE 802.15.4/Zigbee, LoRaWAN, NB-IoT, BLE Mesh, and 6LoWPAN, allowing the proposed routing strategy to operate without modifications to the underlying wireless communication protocols. Consequently, the proposed framework can be integrated into existing IoT infrastructures while preserving interoperability with standardized networking technologies [8,20]. At the Medium Access Control (MAC) layer, the proposed framework considers practical communication constraints such as duty-cycle limitations, channel contention, packet collisions, available bandwidth, and control-message overhead. Rather than continuously updating routing decisions, the MARL component performs event-driven policy updates triggered by significant changes in residual energy, link quality, or traffic conditions. This strategy reduces unnecessary routing-control traffic, lowers bandwidth utilization, and improves communication efficiency while maintaining reliable packet delivery [11,20]. At the network layer, the proposed MARL-BA-ABC framework performs intelligent cluster-head selection and adaptive multi-hop routing. The BA improves local exploitation by identifying energy-efficient cluster-head candidates, whereas ABC algorithm enhances global exploration to prevent premature convergence. MARL dynamically determines routing paths according to residual energy, node density, traffic load, and wireless link quality. This cooperative optimization enables balanced energy consumption, improved routing reliability, and extended network lifetime under dynamic network conditions [8,11,20]. The computationally intensive optimization and reinforcement-learning training processes are executed at the edge layer, including cluster-head gateways, fog nodes, or edge servers with higher computational capability. Ordinary sensor nodes execute only lightweight routing inference using locally stored routing tables or compact Q-tables received from the gateway. This hierarchical architecture substantially reduces processor utilization, memory requirements, and energy consumption at battery-powered sensor nodes while enabling scalable deployment across large IoT networks [20]. From an implementation perspective, the proposed framework is suitable for deployment on low-power embedded platforms equipped with microcontrollers such as the ARM Cortex-M4, which typically provide 128–512 KB Flash memory and 32–128 KB SRAM. The memory footprint is primarily determined by the local routing table and compact reinforcement-learning data structures, making the framework compatible with contemporary embedded WSN hardware. Since wireless communication remains the dominant source of energy consumption, executing optimization at the gateway level significantly reduces the computational energy overhead imposed on individual sensor nodes [20]. At the application layer, the proposed framework can support a wide range of IoT services, including smart-city monitoring, healthcare systems, industrial automation, precision agriculture, and environmental monitoring, where long-term autonomous operation, reliable communication, and energy-efficient routing are essential. The combination of adaptive routing, distributed optimization, and gateway-assisted reinforcement learning makes the proposed MARL-BA-ABC framework suitable for practical large-scale IoT deployments requiring high scalability, low communication overhead, and prolonged network lifetime [8,11].

## 7. Conclusions

This paper presented a novel Multi-Agent Reinforcement Learning-Assisted Hybrid Bat-Artificial Bee Colony (MARL-BA-ABC) framework for energy-efficient clustering and routing in IoT-enabled wireless sensor networks. The proposed framework integrates Bat Algorithm-based local exploitation, Artificial Bee Colony-based global exploration, and Multi-Agent Reinforcement Learning-based adaptive routing within a unified optimization architecture. Unlike conventional approaches that optimize clustering and routing independently, the proposed framework jointly considers cluster-head selection and routing decisions according to residual energy, communication distance, node density, traffic load, congestion level, and link quality. Experimental evaluation demonstrated that the proposed framework consistently outperformed the selected benchmark algorithms in terms of network lifetime, residual energy preservation, packet delivery ratio, throughput, routing efficiency, and communication latency under the adopted simulation settings. The ablation analysis further showed that the integration of BA, ABC, and MARL provides greater overall performance than any individual optimization component, confirming the complementary contributions of local exploitation, global exploration, and adaptive learning. From a practical perspective, the proposed framework is compatible with existing IoT communication technologies because it operates primarily as a network-layer optimization mechanism while preserving standard communication protocols. The gateway-assisted implementation and event-driven routing-policy updates reduce computational and communication overhead, making the framework suitable for resource-constrained IoT-enabled wireless sensor networks. Nevertheless, several limitations should be acknowledged. The current evaluation is based primarily on simulation experiments conducted under controlled network conditions. Although the framework demonstrated consistent performance improvements across repeated simulation runs, additional validation using real-world wireless sensor platforms, heterogeneous IoT environments, and hardware implementations is required. Furthermore, the computational requirements of reinforcement learning and hybrid optimization may require careful parameter tuning for highly resource-constrained devices.

Future research will focus on implementing the proposed framework on practical IoT hardware platforms, investigating lightweight reinforcement-learning techniques for embedded sensor nodes, extending the framework to mobile and energy-harvesting wireless sensor networks, and evaluating additional deep reinforcement learning approaches such as Deep Q-Networks, Double DQN, and Proximal Policy Optimization under dynamic large-scale IoT deployments.

## Figures and Tables

**Table 1:** Comparative analysis of existing approaches.

Method	Clustering	Routing	Learning	Main Limitation
LEACH	Yes	Yes	No	Static probabilistic cluster-head selection
HEED	Yes	Partial	No	Local optimum convergence
PEGASIS	Partial	Yes	No	High communication delay
PSO	Yes	Yes	No	Premature convergence
ACO	Partial	Yes	No	High computational overhead
BA	Partial	Yes	No	Limited global exploration
ABC	Partial	Yes	No	Slow convergence
GWO	Yes	Yes	No	Parameter sensitivity
HHO	Yes	Yes	No	Performance instability
Q-Learning	No	Yes	Yes	State-space explosion
DQN	No	Yes	Yes	High computational complexity
MARL	Partial	Yes	Yes	No explicit clustering optimization
Proposed MARL-BA-ABC	Yes	Yes	Yes	Improved joint clustering–routing optimization through adaptive learning, global exploration, and local exploitation

**Table 2:** System parameters of the proposed MARL-BA-ABC framework.

Category	Parameter	Symbol	Value	Unit
Network	Number of sensor nodes	N	100–1000	nodes
Network	Deployment area	A	1000 × 1000	$m^{2}$
Network	Base station location	BS	(50, 150)	m
Energy	Initial node energy	$E_{0}$	2	J
Energy	Electronic energy	$E_{elec}$	50	nJ/bit
Energy	Data aggregation energy	$E_{DA}$	5	nJ/bit/message
Energy	Free-space amplifier	$\varepsilon_{fs}$	10	$pJ/bit/m^{2}$
Energy	Multipath amplifier	$\varepsilon_{mp}$	0.0013	$pJ/bit/m^{4}$
Routing	Packet size	l	4000	bits
Routing	Maximum communication rounds	R	5000	rounds
BA	Loudness	A	0.9	agents
BA	Pulse emission rate	r	0.5	agents
BA	Frequency range	$f_{min}-f_{m\times n}$	0–2	agents
ABC	Employed bees	ABC	25	agents
ABC	Onlooker bees	ABC	25	agents
ABC	Scout bee limit	Limit	100	agents
MARL	Learning rate	α	0.1	agents
MARL	Discount factor	γ	0.9	agents
MARL	Exploration rate	ε	0.20	agents
MARL	Minimum exploration rate	$\varepsilon_{min}$	0.01	agents
MARL	Exploration decay factor	λ	0.995	agents

**Table 3:** Ablation analysis of the proposed framework.

Method	FND (Rounds)	HND (Rounds)	LND (Rounds)	PDR (%)	Residual Energy (J)
BA Only	1850	2750	3650	94.6	0.94
ABC Only	1900	2820	3720	95.2	1.01
MARL Only	1980	2910	3840	93.6	0.82
BA + ABC	2030	3010	3980	94.1	0.86
BA + MARL	2100	3150	4150	95.3	0.93
ABC + MARL	2140	3210	4210	95.8	0.97
Proposed MARL-BA-ABC	2200	3350	4450	98.1	1.32

## Data Availability

The simulation datasets, configuration files, generated results, and analysis scripts supporting the findings of this study will be made available in a public repository upon publication. During peer review, these materials are available from the corresponding author upon reasonable request.
